# Global, regional, and national time trends in falls and their predictions: an age-period-cohort analysis of the Global Burden of Disease Study 2021

**DOI:** 10.3389/fpubh.2025.1598507

**Published:** 2025-08-01

**Authors:** Huan Jiang, Ning Sun, Huiqi Yang, Ye Li, Lihong Jiang, Huicui Zhao, Yi Zeng, Chengyang Sun, Chun Xu, Yongqiang Lai, Jia Meng

**Affiliations:** ^1^General Medical Department, Second Affiliated Hospital of Harbin Medical University, Harbin, Heilongjiang, China; ^2^Ningbo College of Health Sciences, Ningbo, Zhejiang, China; ^3^Research Center of Public Policy and Management, School of Health Management, Harbin Medical University, Harbin, Heilongjiang, China; ^4^School of Public Health, Hangzhou Normal University, Hangzhou, Zhejiang, China; ^5^Department of Health & Biomedical Science, College of Health Professions, Texas, TX, United States

**Keywords:** falls, time trends, epidemiology, Global Burden of Disease Study 2021, aging

## Abstract

**Background:**

Falls are a global public health issue with a heavy disease and socioeconomic burden, with an increasing number of risky people. The aim of this study is to explore the global disease burden of falls and to provide a scientific basis for the prevention and management of falls.

**Methods:**

This study used data from the Global Burden of Disease Study 2021 (GBD 2021) to analyse global, regional, and national temporal trends in falls. An age-period-cohort (APC) model was applied to analyse data from 1990 to 2021, with global predictions made through 2046.

**Findings:**

Although the falls mortality of all age groups has shown a slight decline globally over the past three decades (Net drift = 0.84%, 95% CI: −0.92, −0.76), the mortality varying across regional and intra-regional levels by sociodemographic index (SDI) quintiles, gender and older adults have not substantially decreased (local drift above 75 years: >0). The APC model reveals that falls mortality has increased with age, especially after the age of 55 years. The period effects shows an overall downward trend (Rate Ratio: 1.08–0.87). The cohort effect shows a trend of increasing first (RR: 1.02–1.18) and then decreasing (RR: 1.18–0.40). The marked heterogeneity in falls mortality is across higher-SDI regions. Population aging is identified as the primary contributor to changes in global falls mortality rates.

**Conclusion:**

Falls among older adults remain a persistent global issue, with significant regional and intra-regional disparities in falls mortality. These findings underscore the urgent need for age- and region-specific interventions to address this public health challenge.

## Introduction

Falls are a serious public health issue (1). Even non-injurious falls can negatively affect people’ quality of life and aging trajectories (2). The most severe consequences of injurious falls are hip fractures and brain injuries (3–6), which can lead to significant physical and mental harm, and impose a substantial socioeconomic burden on people, families, and society. Falls are a common health problem in older adults. Approximately 28–35% older adults experience falls every year (1, 2, 6–8). Understanding the full impact of falls and strengthening prevention and intervention efforts have become increasingly vital for maintaining the health and well-being of the older adults (8, 9).

There was a lack of direct investigations and concrete analysis into the global disease burden of falls in injury research. Compared with previous studies, the novelties of this study were reflected in the content, method and schemes (10–19). In terms of content, this study has supplemented and improved the data volume, regional scope, time span, age group and influencing factors. We used the latest publicly available global-level databases to more accurately reflect current health conditions and trends. This study scrutinised the temporal trends and projections of the disease burden from falls at global regional and national levels for multi-scale space under the full time chain from 1990 to 2046. This study further subdivides the age group, which can more accurately analyze the disease burden of different age groups. It also explored the impact of various factors, including age, period, cohort, gender, population growth, aging, and epidemiological changes on these trends. In terms of methods, this study analyzed the age, period, and cohort effects of the time trend of death from falls based on an age-period-cohort (APC) model in terms of methods. On the schemes side, this study integrated the falls deaths data at the global level and all age groups to analyze the time trend of falls by sociodemographic index (SDI) quintiles and gender from 1990 to 2021 and its global prediction by 2046. On the contribution side, this study enabled policymakers to prioritize the locations and the age groups with the most contribution to the disease burden of falls. This study also provided key information on the epidemiological patterns of falls. This study served as a valuable reference to guide falls prevention and management strategies.

## Methods

### Data sources

Data for this study were sourced from the Global Burden of Disease Study 2021 (GBD 2021) database^1^ (11, 20, 21). We selected ‘all countries or regions (204)’ or ‘all GBD regions (11)’ as the location based on Socio-demographic Index (SDI) quintiles. ‘Falls’ was selected as the cause, with ‘Deaths’ as the primary measure. Additional metrics included ‘Incidence’, ‘Number’, ‘Percentage’, and ‘Rate’ as additional metrics from the database. Gender categories were ‘Female’, ‘Male’, and ‘Both’. Both include male and female. Regarding age, we included both ‘All ages’ and ‘Age standardised’ groups, and further stratified the population into 18 age groups ranging from ‘<5 years’ to ‘85 + years’. The prediction analysis also referenced categories from ‘0–14 years’ or ‘65 years’ to ‘95 + years’. Furthermore, all the countries were categorised into SDI quintiles based on their 2021 SDI values. The economic development levels of each country and region were classified into five quintiles: low, low-middle, middle, high-middle, and high SDI (11, 22). Finally, we selected data from 1990 to 2021. The year in which the analysis was conducted and the results were obtained while adhering to all the aforementioned criteria.

### Data management

The main parameters of the Age–period–cohort (APC) model included both the net drift and local drift. The net drift represented the overall annual percentage change in falls mortality from 1990 to 2021, considering nonlinear age and cohort effects. Local drift represented the annual percentage change in the mortality rates from falls for each age group over time (23–25). We also used indicators such as all-age mortality (crude mortality rate, CMR), age-standardised mortality rate (ASMR), age-standardised incidence rate (ASIR), and estimated the annual percentage change, in which the standardised data were based on the global age-standardised population weights from the GBD 2021 database.

### Statistical analysis

This study utilised an age-period-cohort (APC) model framework to analyse temporal trends in global falls data from 1990 to 2021, as documented in the GBD 2021 database. The analysis was conducted by estimating the impacts of age, period, and cohort on global falls deaths. This analytical methodology has been extensively applied in descriptive epidemiological studies of various chronic diseases. The APC model, a specialized Poisson loglinear model. This study mainly uses Age Period Cohort Analysis Web Tool to calculate. APC analysis can inform registry-based studies of cancer incidence and mortality. This tool contains a panel of easy-to-interpret estimable APC functions and corresponding Wald tests in R code that can be accessed through a user-friendly Web tool. The APC model was fitted using the APC web page analysis tool,^2^ with parameters estimated via the Wald χ2 test embedded on the web page. The significance level was set at α = 0.05, and the outcome yielded a *p*-value of < 0.05 (23–25). We applied variance decomposition analysis to quantify the overlapping contributions of different factors affecting falls populations to the overall variance in their mortality rates. An aging, population growth, and epidemiological changes breakdown analysis of falls mortality enables the quantification of these factors’ contributions to the aggregate impact (26). In addition, this study used the Nordpred software package of R to predict the global disease burden in the whole and the older adult population by 2046. The projected population data were derived from the World Population 2024 provided by the Population Division of the Department of Economic and Social Affairs of the United Nations Secretariat Outlook Revised UN Official Population Estimates and Projections.^3^ All analyses were performed in R (version 4.4.0), except for the world map section, using ArcGIS (version 10.8.)

### Results

Compared to 1990, falls were the leading cause of unintentional injury mortality among the global older adult population, and by 2021, falls had topped the leading cause of death from unintentional injuries in the entire population worldwide, except for road injuries (Supplementary Figure S1). According to the data of different SDI regions in 2021, the proportion of deaths from falls in all SDI regions was significantly higher in 2021 than in 1990, with the largest increase observed in high-SDI regions, where falls accounted for more than 50% of the deaths (Supplementary Figure S2).

### Global, regional and national trends in falls mortality, 1990–2021

The results showed that over the past 30 years, the global population had increased from 5.3 billion (95% Uncertainty Interval [UI]: 5.2–5.5) to 7.9 billion (95% UI: 7.7–8.1), and the number of deaths from falls increased by 96.9% from 4,07,768 to 8,02,803 persons. In 2021, the global crude mortality rate of falls reached 10.71 per 100,000 (95% UI: 8.64, 10.08), which was a substantial increase of 33.07% (95% UI: 29.01, 31.28) compared to 1990 (7.65 per 100,000, 95% UI: 6.7, 8.44). However, falls ASMR decreased from 10.90 (95% UI: 9.68, 11.81) per 100,000 in 1990 to 9.94 (95% UI: 8.43, 10.84) in 2021 (percent change of rate: -8.79; 95% UI: −12.97,-8.19). The overall annual percentage change (net drift) of falls mortality among all age groups estimated from the APC model was 0.84% (95% Confidence Interval [CI]: −0.92, −0.76) showing a decreasing trend over the past 30 years. At the regional level, it was found that the specific values (local drift) of falls mortality varied from region to region according to the SDI quintiles. Falls mortality was negatively associated with the SDI. This also indicated a more pronounced reduction in falls mortality risk in regions with a higher SDI status, while regions with a lower SDI tended to demonstrate a slight decline in falls mortality. Collectively, the falls mortality trends indicated by these conventional indicators were uneven between countries and regions according to the SDI quintiles. The data indicate that the direction of change in falls mortality presented by the ASMR and its change were fully consistent with the net drift derived from the APC model. Among the 21 GBD regions between 1990 and 2021, falls ASMR increased in only six regions with the highest increase in Southern Latin America (net drift = −1.5%;95% CI: −1.8,-1.2). In addition, most regions experienced a decline in falls motility, with the largest declines in Eastern Europe, Central Europe, and East Asia. Additional details are provided in Tables 1, 2.

**Table 1:** Trends in falls mortality across socio-demographic Index quintiles, 1990–2021.

Characteristic	Global		High SDI		High-middle SDI		Middle SDI		Low-middle SDI		Low SDI	
	1990	2021	1990	2021	1990	2021	1990	2021	1990	2021	1990	2021
Population												
Number, n × billion(95% UI)	5.33 (5.23 to 5.44)	7.89 (7.67 to 8.13)	0.88 (0.86 to 0.90)	1.09 (1.06 to 1.13)	1.06 (1.03 to 1.10)	1.30 (1.25 to 1.36)	1.72 (1.67 to 1.77)	2.45 (2.35 to 2.54)	1.16 (1.12 to 1.20)	1.92 (1.82 to 2.02)	0.50 (0.49 to 0.51)	1.12 (1.07 to 1.17)
Deaths												
Number, n × 100,000 (95%UI)	4.08 (3.57 to 4.50)	8.03 (6.82 to 8.74)	9.70 (0.76 to 1.11)	1.76 (1.50 to 1.90)	0.39 (0.31 to 0.46)	1.25 (0.97 to 1.48)	1.15 (0.99 to 1.29)	2.28 (1.79 to 2.60)	0.81 (0.75 to 0.85)	1.98 (1.69 to 2.20)	0.75 (0.69 to 0.84)	0.75 (0.63 to 0.88)
Percent change, 1990–2021, % (95%UI)	47.23 (40.82 to 54.22)		42.40 (40.88 to 43.83)		49.76 (39.79 to 60.58)		67.25 (55.17 to 81.05)		41.29 (33.96 to 50.04)		25.67 (16.21 to 36.81)	
APC model estimates												
Net drift, % per year	−0.84(−0.92 to −0.76)		−1.02(−1.12 to −0.92)		−1.58(−1.71 to −1.46)		−0.91(−0.99 to −0.83)		−0.67(−0.81 to −0.54)		−0.82(−0.97 to 0.68)	
All-age mortality rate												
Rate, per 100,000	7.65 (6.70 to 8.44)	10.17 (8.64 to 10.08)	9.23 (8.5 to 9.62)	16.05 (13.75 to 17.33)	7.1 (6.52 to 7.91)	9.60 (7.44 to 11.34)	6.68 (5.75 to 7.48)	9.32 (7.31 to 10.6)	8.33 (6.58 to 9.51)	10.29 (8.79 to 11.44)	7.73 (6.10 to 9.51)	6.75 (5.63 to 7.85)
Percent change, 1990–2021, % (95%UI)	−0.49(−4.82 to 4.23)		14.48 (13.26 to 15.63)		22.14 (14.01 to 30.96)		17.68 (9.19 to 27.39)		−14.59(−19.01 to −9.29)		−43.62(−47.87 to −38.62)	
Age-standardised mortality rate												
Rate, per 100,000	10.90 (9.68 to 11.81)	9.94 (8.43 to 10.84)	7.8 (7.15 to 8.14)	7.3 (6.40 to 7.81)	8.72 (7.91 to 9.58)	7.07 (5.51 to 8.32)	11.78 (10.14 to 12.82)	9.9 (7.75 to 11.24)	16.38 (13.6 to 18.99)	15.81 (13.54 to 17.69)	18.55 (15.75 to 21.74)	17.40(14.7 to 20.34)
Percent change, 1990–2021, % (95%UI)	−24.55(−27.78 to −21.04)		−29.08(−33.57 to −24.21)		−30.91(−31.70 to −30.12)		−25.99(−31.41 to −20.00)		−18.77(−23.38 to −13.631)		−16.50(−20.57 to −11.68)	
EAPC												
EAPC, 1990–2021, No.(95%CI)	0.95 (0.23 to 1.68)		1.8 (1.67 to 1.93)		1.02 (0.03 to 2.01)		1.14 (0.09 to 2.21)		0.72 (0 to 1.46)		−0.41 (−1.47 to 0.67)	

SDI=Socio-demographic Index; APC = age-period-cohort. EAPC = estimated annual percentage change. Net drifts are estimates derived from the APC model and denotes overall annual percentage change in falls mortality. Parentheses for all GBD health estimate indicate 95% uncertainty intervals; parentheses for net drift and EAPC indicate 95% confidence intervals.

**Table 2:** Trends in falls mortality across 21 GBD regions, 1990–2021.

Region	1990		2021		APC model estimates
	All-age mortality rate, No. (95% UI)	ASMR, No. (95% UI)	All-age mortality rate, No. (95% UI)	ASMR, No. (95% UI)	Net drift, % per year (95%CI)
Andean Latin America	4.7 (4.2 to 5.4)	7.4 (6.5 to 8.5)	5.9 (4.7 to 7.1)	6.5 (5.2 to 7.9)	−0.8 (−1 to −0.6)
Australasia	5.7 (5.2 to 6.1)	5.5 (4.9 to 5.9)	16.6 (13.8 to 18.2)	8.2 (6.9 to 8.9)	0.7 (0.2 to 1.2)
Caribbean	6.4 (6 to 6.7)	9.7 (9.1 to 10.2)	11.9 (10.5 to 13.2)	10.1 (9 to 11.2)	−0.4 (−0.7 to −0.1)
Central Asia	5.4 (5.1 to 5.6)	6 (5.7 to 6.2)	3.4 (3.0 to 3.8)	3.7 (3.3 to 4.1)	−1.3 (−1.4 to −1.1)
Central Europe	15 (14.3 to 15.5)	15 (14.2 to 15.6)	13.9 (12.6 to 14.9)	7.6 (6.9 to 8.1)	−2.4 (−2.5 to −2.2)
Central Latin America	6.3 (6.1 to 6.4)	11.2 (10.8 to 11.5)	5.2 (4.7 to 5.7)	5.4 (4.8 to 5.9)	−2.3 (−2.4 to −2.2)
Central Sub-Saharan Africa	3.9 (3.1 to 5.3)	10.8 (8.5 to 14.4)	3.5 (2.5 to 5)	10.5 (7.8 to 15.1)	−0.3 (−0.5 to −0.1)
East Asia	6.2 (5.3 to 8.2)	10 (8.7 to 12.9)	9.9 (6.5 to 12.8)	8.5 (5.5 to 10.9)	−0.7 (−0.8 to −0.6)
Eastern Europe	6.9 (6.7 to 7)	6.5 (6.3 to 6.6)	8.5 (7.9 to 9.1)	5.9 (5.5 to 6.3)	−1.9 (−2.1 to −1.7)
Eastern Sub-Saharan Africa	5.9 (4.6 to 6.8)	17 (14.4 to 20.1)	5 (4.2 to 5.8)	15 (12.8 to 17.7)	−0.9 (−1 to −0.7)
High-income Asia Pacific	4.8 (4.4 to 5.3)	4.6 (4.2 to 5.1)	11.7 (9.5 to 13.1)	3.8 (3.3 to 4.3)	−2.6 (−2.7 to −2.4)
High-income North America	5.6 (5 to 5.9)	4.5 (4 to 4.7)	15.7 (13.5 to 17)	8.2 (7.1 to 8.8)	0.7 (0.5 to 0.8)
North Africa and Middle East	4.4 (3.5 to 5.6)	7.7 (6.3 to 9.3)	3.8 (3.2 to 4.4)	5.7 (4.8 to 6.7)	−1 (−1.1 to −0.9)
Oceania	3.3 (2.4 to 4.4)	7.9 (5.4 to 10.6)	4 (2.4 to 5.7)	7.9 (4.8 to 11.3)	0.3 (−0.3 to 0.9)
South Asia	10.7 (7.9 to 12.5)	22.8 (17.5 to 26.9)	14.2 (11.6 to 15.9)	21.2 (17.3 to 23.8)	−0.8 (−1 to −0.6)
Southeast Asia	6.7 (5.5 to 7.6)	12.6 (9.6 to 14.8)	8.3 (6.7 to 9.5)	10.4 (8 to 11.8)	−0.9 (−0.9 to −0.8)
Southern Latin America	4.9 (4.6 to 5.1)	5.8 (5.4 to 6.1)	5.2 (4.7 to 5.5)	4 (3.6 to 4.2)	−1.5 (−1.8 to −1.2)
Southern Sub-Saharan Africa	2.1 (1.7 to 2.4)	3.8 (3 to 4.4)	2.2 (1.9 to 2.7)	3 (2.6 to 3.8)	−0.6 (−0.9 to −0.3)
Tropical Latin America	5.2 (4.9 to 5.3)	8.5 (7.9 to 8.8)	9.6 (8.6 to 10.2)	9 (8 to 9.6)	−1 (−1.1 to −0.9)
Western Europe	14.2 (12.9 to 14.9)	9.9 (9 to 10.4)	20.8 (17.5 to 22.5)	7.8 (6.8 to 8.4)	−1.5 (−1.6 to −1.3)
Western Sub-Saharan Africa	6.2 (5.3 to 7.5)	14.7 (12.4 to 17.8)	4.8 (3.9 to 5.6)	13.4 (11.4 to 15.8)	−0.6 (−0.7 to −0.5)

GBD = Global Burden of Disease; APC = age-period-cohort. ASMR = Age-standardised mortality rate. Age-standardised mortality rate is computed by direct standardisation with global standard population in GBD 2021. Net drifts are estimates derived from the APC model and denotes overall annual percentage change in falls mortality. UI: Uncertainty interval. CI = Confidence interval.

**Figure 1:**
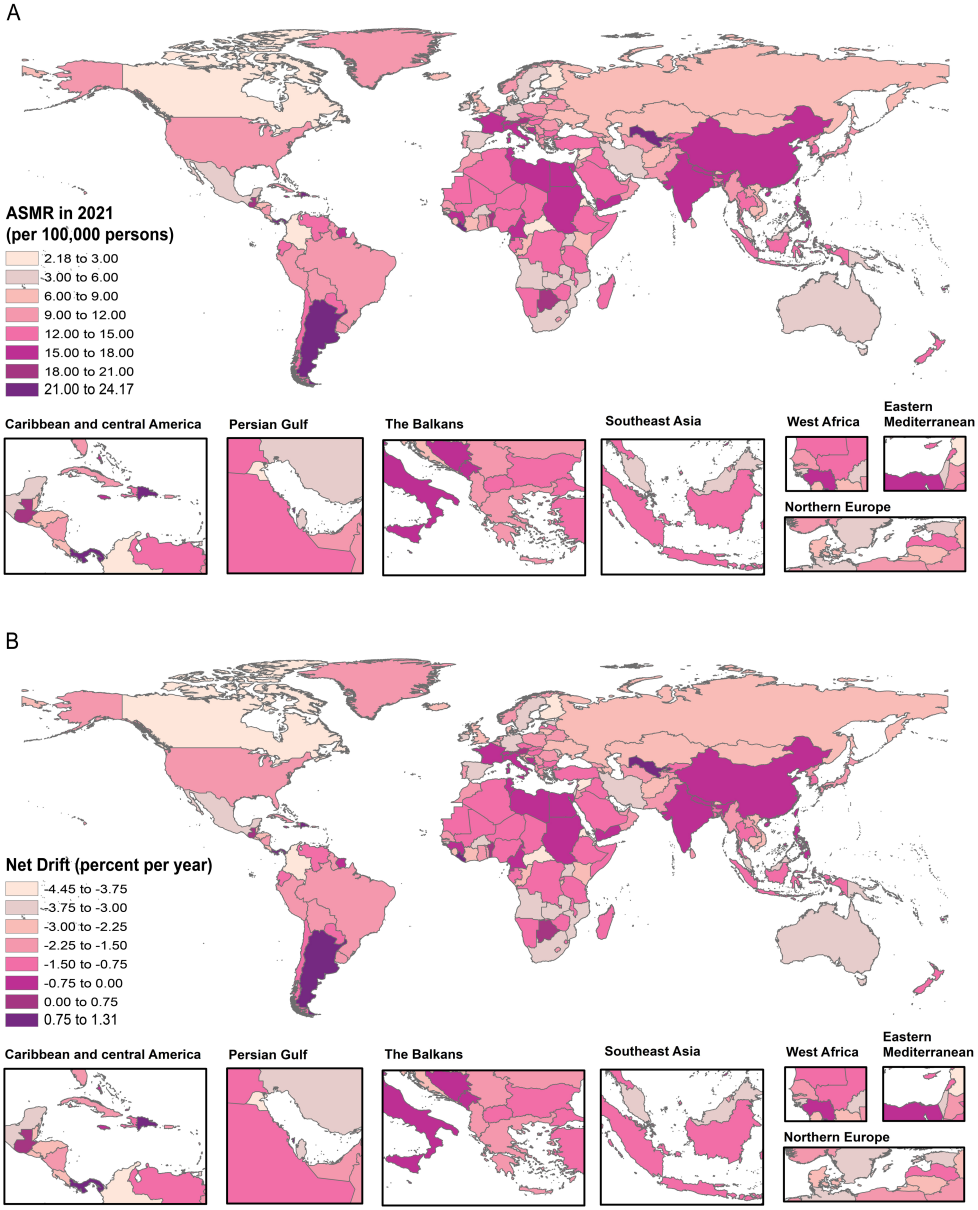
The global disease burden map of falls in 204 countries and territories. The ASMR for falls in 2021 **(A)**. The net drift of falls based on the APC model from 1990 to 2021 **(B)**.

At the national level, among 204 countries and regions worldwide, the highest ASMR of falls in 1990 was observed in Europe and other regions with higher SDI values, such as Hungary (33.3/100,000, 95% UI: 30.9, 35.4) and Czechia (26.2/100,000, 95% UI: 24.3, 28.0). However, most of the higher ASMR of falls in 2021 were countries with lower SDI, such as India (24.2 per 100,000, 95% UI: 19.7, 27.3). In addition, in the past 30 years, the highest increase in ASMR of falls mostly occurred in countries with higher SDI, such as Australia (net drift = 0.84%; 95% CI: 0.26, 1.14), Europe, United States of America (net drift = 0.67%; 95% CI: 0.53, 0.81), and Canada (net drift = 0.56%; 95% CI: 0.13, 0.98). Furthermore, favourable reductions in fall mortality were found in countries with higher SDI status as well, especially in Europe, such as Hungary and Czechia, which declined most obviously with an estimated annual percentage change of 4.45% (net drift, 95% CI: −4.89, −4.02) and 3.20% (net drift, 95% CI: −3.67, −2.74). Additional details are provided in Figure 1 and Supplementary Table S1.

### Results on APC model

**Figure 2:**
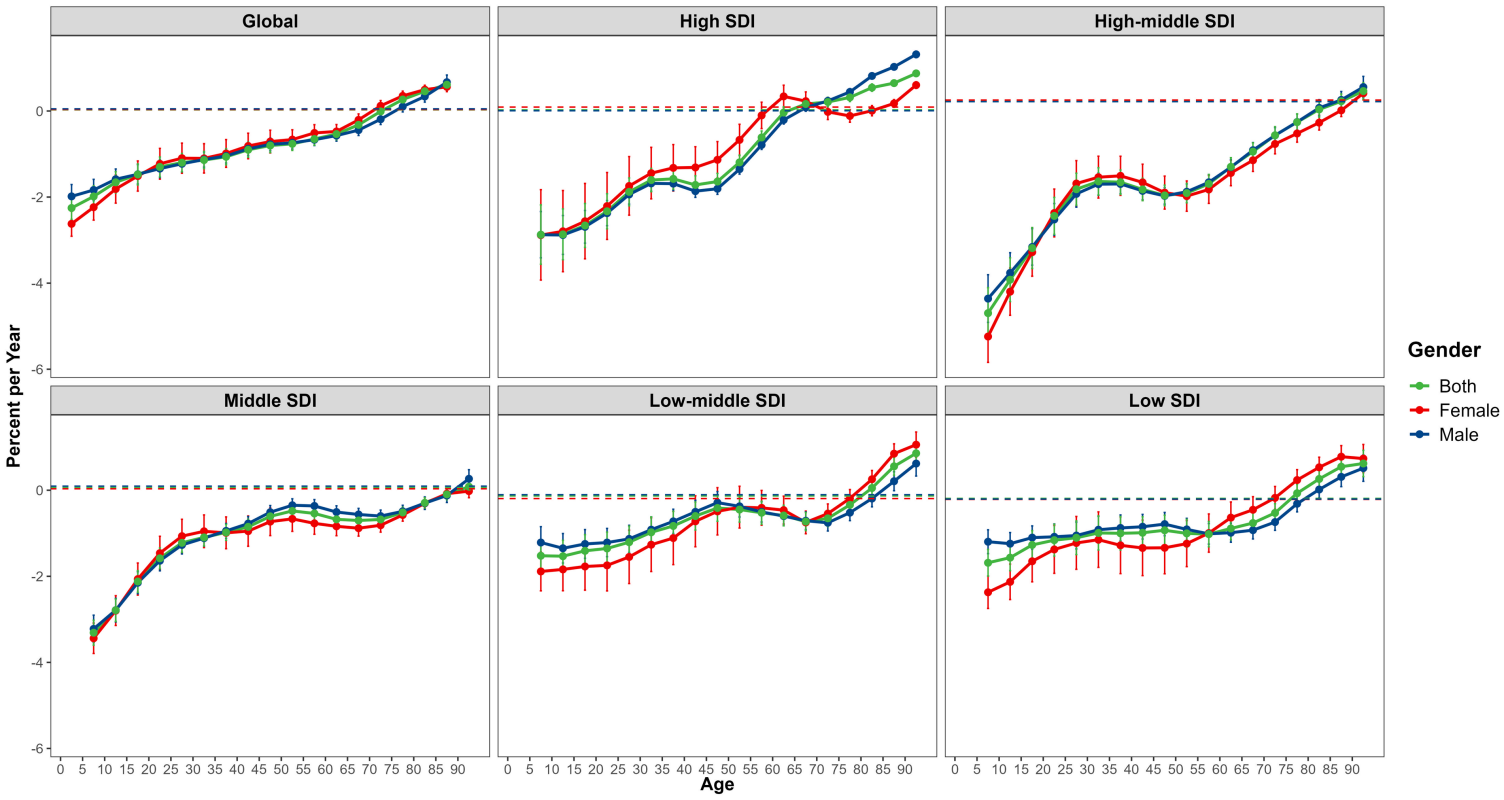
The local drifts of falls mortality for 18 age groups (from <5 years to 85 + years) by SDI quintiles and gender from 1990 to 2021.

The annual percentage change (local drift) in falls mortality varied among different age groups according to the SDI quintiles (Figure 2). Falls mortality increased with age, particularly in older adults (>75 years), with a marked upward trend (local drift > 0 percent per year). Overall, the age at which falls mortality began to increase varied across regions. Falls mortality began to rise at approximately 65 years in the high-SDI regions, 80 years in the high-middle SDI regions, 90 years in the middle-SDI regions, 80 years in the low-middle SDI regions, and 75 years in the low-SDI regions. Notably, except for the population aged > 90 years, the overall falls mortality trend in the middle SDI region showed a decline (Figure 2; Supplementary Figure S3).

Globally, the effects of age on falls mortality show that the burden of falls deaths increases significantly with age, especially among older adults (above 50 years of age), with an obvious upward trend. Among the different SDI regions, the falls mortality curve in the low-SDI region was the steepest across all age groups.

**Figure 3:**
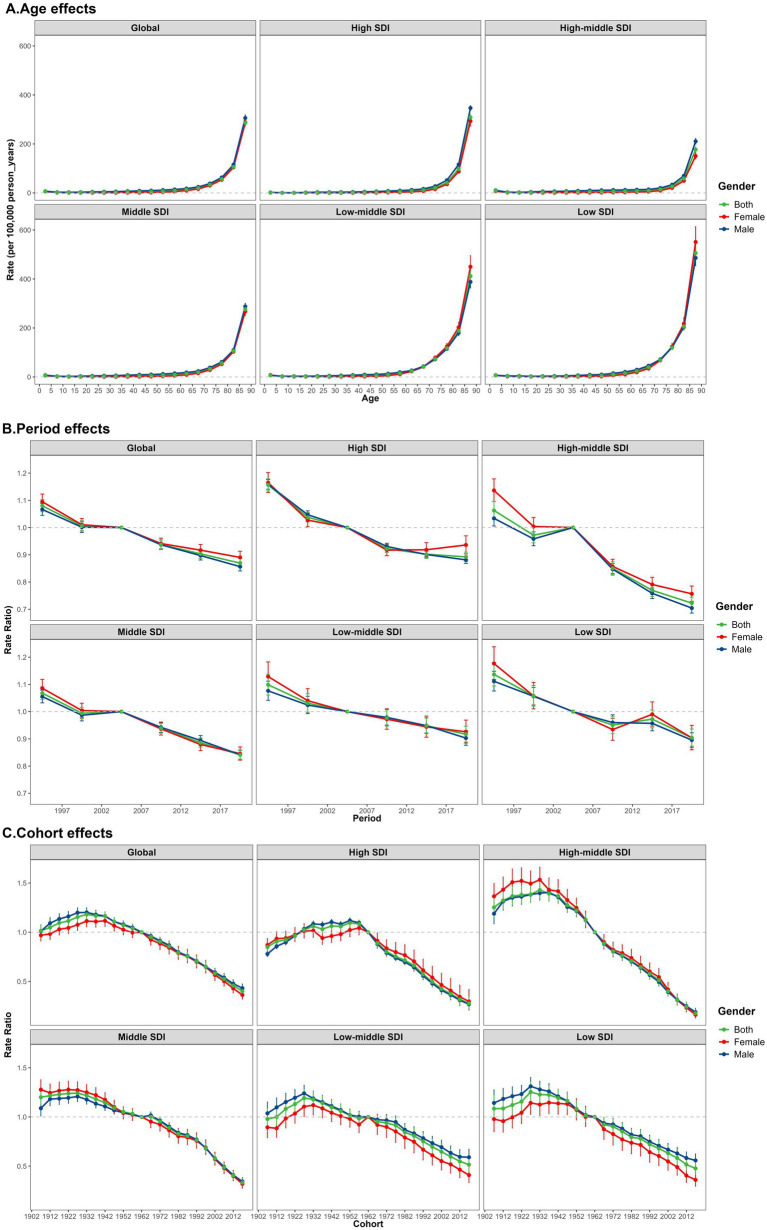
Age-period-cohort modelling: estimation of mortality of falls by SDI quintiles and gender from 1990 to 2021. **(A)** The falls mortality (per 100,000) after adjusting for age effects. **(B)** The falls mortality rate ratio (RR) after adjusting for period effects. **(C)** The falls mortality rate ratio (RR) after adjusting for cohort effects.

The period effects of falls mortality generally showed an overall downward trend across different SDI regions (Rate Ratio [RR]:1.08–0.87). Between 1992 and 1997, and following 2004, the risk of fall-related mortality in the high-middle and middle SDI regions experienced the most significant decline. A brief plateau was observed from 1997 to 2004, after which the decline resumed. In contrast, in regions with a high SDI, the reduction in period risk was more favourable between 2004 and 2012. In regions with a lower SDI status, the overall reduction in the burden of fall-related deaths was not significant. From 2007 to 2017, low-SDI regions experienced two turning points, followed by a sustained decline in falls deaths. Globally, the cohort effects revealed that the overall risk of falls mortality initially increased (RR: 1.02–1.18), before reversing in the 1932 birth cohort (RR = 1.18, 95% CI: 1.14, 1.23), which exhibited a downward trajectory (RR: 1.18–0.40). The most pronounced increases and decreases in the cohort effects of falls mortality were observed in regions with a higher SDI. Additionally, the cohort risk in the middle SDI regions increased gradually, generally indicating a stable downward trend. This study also found that improvements in falls mortality occurred earlier in regions with a lower SDI. In high SDI regions, the falls mortality rate ratio displayed a fluctuating upward trend between the 1927 and 1957 birth cohorts (RR > 1), with the peak risk observed in the 1952 birth cohort (RR = 1.10, 95% CI: 1.06, 1.13). Across different SDI regions, the relative cohort risk for individuals born in the 2017 cohort varied from 0.95 (95% CI: 0.89–1.02) in low-middle SDI regions to 0.18 (95% CI: 0.15–0.22) in high SDI regions (Figure 3; Supplementary Figure S4).

### Sex differences and decomposition analysis results

There were gender-related differences in the effects of age on falls mortality from 1990 to 2021. With the increase of age, the gender difference of falls mortality showed a cross trend, and in the older group, females were higher than males. Gender differences between different regions showed different results. (Figures 2, 3) Excluding the impact of age factors, there are certain gender disparities in the risk of global fall-related mortality according to the SDI quintiles in 2021. Generally, males had a higher risk of falls mortality than females (Supplementary Figure S5). Furthermore, this study conducted a decomposition analysis of the burden of population growth, aging, and epidemiological changes in falls deaths using SDI quintiles. The findings indicated that population growth and aging are major drivers of the increase in global falls mortality from 1990 to 2021, with aging being the predominant cause. Notably, the corresponding burden was most pronounced in the high-SDI regions (Supplementary Figure S6).

### Predictions of falls

Based on the APC model results, the data on falls among all age groups (Supplementary Figure S7) and older age groups (65–69 to 95 + years) (Supplementary Figure S8) were chosen for the predictive modelling of the global disease burden of falls. The projections of global morbidity and mortality for falls in different age groups by 2046 reveal that falls morbidity (ASIR) is expected to increase and mortality (ASMR) will decrease in all age groups, whereas the mortality of falls in the older adults will increase by 2046. To better understand the burden of falls deaths, we divided the data according to gender and analysed the predicted changes in falls morbidity and mortality in both males and females. The overall morbidity and mortality of falls across all age groups is projected to be higher in males than in females by 2046. However, the opposite is true for falls mortality among the older adults.

## Discussion

Falls constituted a major global public health concern, as they were the primary cause of death from unintentional injury (1, 10, 27). Our study conducted a thorough analysis and forecast of the temporal trends in falls fatalities from 1990 to 2021, examining the global, regional, and national levels. We compared the burden of falls deaths across various SDI and GBD regions and countries. The findings indicated that the global mortality rate for falls had generally declined. However, disparities in falls fatality rates persisted among different SDI regions, as well as at the regional and national levels.

The APC model indicated that trends in falls mortality varied across different SDI regions, accounting for the effects of age, period, and cohort. Age effect showed that the global death burden from falls increased with age, particularly in the older adults. The period effects on falls mortality exhibited a general decline, whereas the cohort effects increased before falling. Notably, favourable reductions and unfavourable increases in falls mortality were predominantly observed in regions with higher SDI values. However, this pattern was not as evident in regions with lower SDI values. Significant temporal trends in falls incidents predominantly manifested in regions with higher SDI values, indicating considerable intra-regional heterogeneity. This phenomenon was most pronounced in Europe. According to this study and previous relevant evidence, Europe has always been a region with a high burden of falls deaths since 1990, particularly in the Netherlands. Different countries and regions have differences in accurate data management and reporting practices. In this study, it was adjusted for age for all age groups since Europe may have an older population as compared with other regions, and therefore more falls (4, 10, 13, 14, 27–30).

This phenomenon has prompted questions regarding the underlying causes of the temporal variations in falls mortality. It has been considered that the temporal trend changes in falls mortality were likely influenced by complex factors (e.g., age, gender, economic level, and low education level) (1, 3, 6, 7, 31–36). This study conducted a longitudinal analysis of the influencing factors of changes in the time trends of falls deaths. Moreover, the current results confirmed that the risk of death from falls in the entire population was associated with age and gender. In terms of gender, variations in age, period, and cohort effects were observed among the different SDI regions. Apart from age, males exhibit a higher mortality rate due to falls than females.

The findings of the decomposition analysis indicated that this phenomenon may stem from the significant impact of demographic structure alterations influenced by a combination of population growth and an aging population, particularly in regions with a higher SDI value. Aging was the predominant cause, as supported by numerous studies (1, 3, 17, 34). Moreover, the change for falls mortality in higher SDI regions in the past 30 years varied from national level, such as some countries or territories (e.g., Australia in Europe, United States of America (USA) and Canada, etc) with faster increase and other ones (e.g., Czechia in Europe) with bigger decrease (1, 3, 17, 34). The findings of these analyses provide further support for the complex factors underlying the intra-regional heterogeneity of falls mortality. This heterogeneity is reflected in the fact that some countries (e.g., Czechia) show a significant decline in falls mortality in regions with high SDI value. This phenomenon may be attributed to the fact that improvements in education and economic levels typically lead to increased health awareness and the development of healthcare infrastructures or public facilities, such as in USA (17). Consequently, regions with a higher SDI may have more effective measures to prevent fatal falls. Conversely, regions with lower SDI status often face challenges in this regard. Therefore, we might infer that a higher percentage of older adult males in lower SDI regions engaging in hazardous occupations continues to contribute to the higher mortality rate from falls. Further investigation is warranted to understand the factors driving the changes in falls mortality in regions with higher SDI value, as these trends are not easily explained.

## Conclusion

This study facilitated the dynamic tracking of epidemiological trends in falls over time, across different geographical regions and genders. It revealed a comprehensive character of global, regional, and national temporal trends in falls from 1990 to 2021, along with projections. Based on our analysis and projections, the incidence of falls is expected to increase across all age groups, while the mortality rate from falls is expected to decrease. However, among the older adult, the mortality rate from falls is expected to increase with age on age effect based on APC model. Aging was one of the main reasons for the temporal trend of falls mortality. The greater emphasis should be placed on reducing the incidence of falls, particularly among older individuals, to mitigate the damage caused by falls. The APC model findings also suggest a significant disparity in falls mortality across regions with higher SDI values, indicating variations in the assessment and prevention strategies for falls that affect the older population in these regions on period and cohort effect. Therefore, falls among older adults remain a persistent global challenge, with notable regional and intra-regional disparities in mortality rates, necessitating targeted preventive strategies tailored to local demographic and socioeconomic contexts (4, 9, 17, 32–36).

## Limitations

First of all, the data we use are from the GBD database, which integrates data from multiple sources, including national and regional reports and publications. Different countries and regions have differences in data management, diagnostic criteria and reporting practices. For example, individuals or family members are often reluctant to report falls, which may affect the accuracy of the results (4, 37). Secondly, falls are affected by a variety of complex factors, but due to the limitations of database data, this study only explored some of these factors, including age, period, cohort, gender, population growth, aging and epidemiological changes. Third, there is a time lag in GBD data, so that our analysis is limited to the period from 1990 to 2021.

The future direction of this study is to make up for the limitations of single data source by incorporating data from multiple sources. Meanwhile, Standardized data calibration methods to improve the accuracy and reliability of the study. This study will be devoted to exploring a path of the real impact factors and multiple risk factors of falls. In addition, we will use data analysis techniques such as machine learning and artificial intelligence to explore the interaction path of multiple risk factors for falls.

Despite these limitations, the consistency of our results with previous population-based and analytical epidemiological studies enhances the validity of our results. Therefore, real world research is needed to further verify our conclusions.

## Data Availability

Publicly available datasets were analyzed in this study. This data can be found here: data is publically available at Global Health Data Exchange (GHDx) online website (http://ghdx.healthdata.org/gbd-results-tool).

## Footnotes

^1^ http://ghdx.healthdata.org/gbd-results-tool

^2^ https://analysistools.cancer.gov/apc/

^3^ https://population.un.org/wpp/
