# Supplementary material for: Global, regional, and national time trends in falls and their predictions: an age-period-cohort analysis of the Global Burden of Disease Study 2021
Source: Front Public Health. 2025 Aug 1;13:1598507. doi: 10.3389/fpubh.2025.1598507 (PMC12354658; doi:10.3389/fpubh.2025.1598507)
Supplement: Supplementary file 1 [file Data_Sheet_1.pdf]

Supplementary Table and figure

Table 1S. Trends in falls mortality across 204 countries, 1990-2021.

Table 1S. Trends in falls mortality across 204 countries, 1990-2021.					
Region	1990		2021		APC model estimates
	All-age mortality rate, No.(95%UI)	ASMR, No.(95%UI)	All-age mortality rate, No.(95%UI)	ASMR No.(95%UI)	Net drift, % per year.(95%CI)
Afghanistan	12.2 (8.6,16.7)	18.3 (13.9,24.2)	7 (5.3,9.2)	15.3 (11.4,21.2)	-0.6 (-0.3,-1)
Albania	2.2 (1.8,2.9)	3.5 (2.8,4.9)	3.3 (2.4,4.9)	2.4 (1.8,3.4)	-1.3 (0.1,-2.7)
Algeria	3.9 (3.1,4.9)	7 (5.2,9.3)	3.6 (2.7,5)	5.3 (4,7.6)	-1.3 (-1,-1.6)
American Samoa	3.5 (3,4.3)	8.3 (7,10.2)	6 (4.7,7.2)	7.9 (6.2,9.5)	0 (7.4,-6.9)
Andorra	7.3 (5.2,10.2)	9.3 (6.7,12.5)	12 (8.5,16.5)	5.8 (4.1,8)	-1.7 (6.9,-9.5)
Angola	4.4 (3.3,6.4)	11.8 (9.4,14.8)	3.4 (2.5,4.5)	11.3 (8.5,14.8)	-0.7 (-0.3,-1.1)
Antigua	3.1 (2.9,3.4)	3.2 (3,3.5)	3.8 (3.6,4.1)	4 (3.7,4.2)	0.2 (11.4,-9.8)
Argentina	4.7 (4.4,4.9)	5.4 (5,5.8)	3.9 (3.5,4.2)	3.2 (2.9,3.4)	-1.8 (-1.4,-2.1)
Armenia	6 (5.5,6.4)	6.7 (6.2,7.2)	3.8 (3.3,4.3)	3 (2.7,3.4)	-2.9 (-2,-3.8)
Australia	5.5 (4.9,5.8)	5.3 (4.7,5.7)	17.3 (14.4,19.1)	8.3 (7,9.1)	0.8 (1.4,0.3)
Austria	18.6 (17,19.7)	12.7 (11.6,13.4)	21.4 (18.2,23.4)	9 (7.8,9.7)	-1.5 (-0.9,-2.2)
Azerbaijan	3.7 (3,4.5)	4.1 (3.3,4.8)	2.1 (1.4,3.1)	2.2 (1.6,3.1)	-1.9 (-1.3,-2.5)
Bahamas	2.5 (2.3,2.7)	4.3 (3.9,4.6)	1.8 (1.4,2.2)	4 (3.1,4.8)	0.5 (4.4,-3.3)
Bahrain	2.1 (1.8,2.5)	5.6 (4.2,6.7)	3.2 (2.4,4.5)	4.3 (3.3,6.3)	-2.2 (0.2,-4.5)
Bangladesh	2.9 (2.2,4)	5.3 (3.8,7.1)	6.9 (5.7,8.1)	4.1 (3.4,4.9)	-1.4 (-1.2,-1.7)
Barbados	4.2 (3.9,4.5)	3.6 (3.4,3.9)	13.1 (10.9,15.6)	8.9 (7.4,10.5)	-0.5 (6.1,-6.7)
Belarus	7.4 (7,7.7)	6.8 (6.5,7.1)	23.7 (19.5,26.1)	9.6 (8.3,10.5)	-1.1 (-0.8,-1.5)
Belgium	13.3 (12,14.2)	9.3 (8.4,9.9)	3.1 (2.7,3.4)	4.8 (4.2,5.3)	-0.6 (0,-1.2)
Belize	1.9 (1.8,2.1)	3.4 (3.1,3.6)	4.7 (3.5,6)	12.8 (9.8,16.1)	0.5 (5.5,-4.2)
Benin	6.7 (5.5,8.1)	15.2 (12.3,18.7)	8.8 (7.5,10.4)	3.7 (3.2,4.4)	-0.8 (-0.1,-1.4)
Bermuda	4.4 (4.1,4.7)	4.8 (4.5,5.1)	15.1 (7.5,22.9)	20.7 (10.4,31.4)	-0.9 (12.9,-13)
Bhutan	8.1 (4,11.9)	22.4 (11.3,33.4)	4.8 (3.8,6.1)	4.7 (3.7,5.9)	-1 (0.5,-2.5)
Bolivia	6.2 (4.7,8.4)	10.5 (8.1,14.2)	3.9 (2.5,5.3)	2.3 (1.5,3)	-1.1 (-0.7,-1.6)
Bosnia and Herzegovina	2.8 (1.9,3.6)	3.3 (2.2,4.2)	2 (1.4,2.7)	3.3 (2.4,4.4)	-1.6 (-0.3,-3)
Botswana	2.3 (1.6,3.2)	5.3 (3.6,7)	9.8 (8.8,10.4)	9.1 (8.1,9.7)	-1.9 (-0.3,-3.3)
Brazil	5.2 (5,5.4)	8.6 (8,8.9)	4.2 (3.5,4.9)	6.1 (5,7.1)	-1 (-0.9,-1.1)
Brunei	4 (3.2,5.1)	7.9 (6.4,9.8)	8.4 (7.4,9.7)	4.9 (4.3,5.6)	-0.8 (1.9,-3.4)
Bulgaria	7.2 (6.6,7.7)	7.1 (6.7,7.6)	6.7 (4.9,8.7)	17.9 (13.7,22.6)	-1.7 (-1.1,-2.3)
Burkina Faso	8.3 (6.9,9.8)	20.3 (16.5,24.6)	4.7 (3.3,6.6)	15.3 (11,21.3)	-0.4 (0.1,-0.8)
Burundi	6.8 (5.2,8.7)	17.6 (13.8,22.7)	13.6 (10.4,17.5)	22.5 (17.3,27.6)	-1 (-0.4,-1.5)
Cambodia	4.5 (3.6,5.5)	6.4 (5.2,7.9)	4.5 (3.4,5.8)	13.1 (10.5,17.1)	-0.8 (2.4,-4)
Cameroon	10 (7.7,12.8)	21.6 (17.8,26.3)	20.4 (17.2,22.1)	9.2 (7.9,10)	-0.3 (0,-0.6)
Canada	6.1 (5.1,7.5)	16.1 (13.4,19.4)	3.7 (2.5,5.7)	11.1 (7.8,15.9)	-0.7 (-0.3,-1.1)
Cape Verde	7.9 (7,8.4)	7 (6.2,7.5)	5 (3.8,6.6)	15.2 (11.8,19.9)	0.6 (1,0.1)

**Central African Republic**	4.9 (3.5,7.2)	13.1 (10,17.5)	7.4 (6.6,8)	5.5 (4.9,5.9)	-0.8 (0,-1.6)
**Chad**	6.5 (5.1,8.4)	14 (10.6,18.4)	10 (6.4,12.9)	8.6 (5.5,11.1)	0.2 (0.8,-0.4)
**Chile**	5 (4.7,5.3)	7 (6.6,7.4)	4.3 (3.6,5)	3.8 (3.1,4.4)	-1.3 (-0.8,-1.7)
**China**	6.2 (5.3,8.2)	10.1 (8.8,13.1)	5.1 (4.2,6.2)	5.8 (4.9,7)	-0.7 (-0.6,-0.8)
**Colombia**	5 (4.8,5.2)	8 (7.6,8.3)	6.9 (4.7,10.1)	13.1 (9.1,19.2)	-2.8 (-2.5,-3.1)
**Comoros**	5.3 (3.9,7)	14.6 (10.7,19.3)	3.7 (2.7,5.2)	9.7 (7.2,13.6)	-1.1 (1.2,-3.4)
**Cook Islands**	4.3 (3.1,6.4)	12.1 (9,16.6)	5.5 (3.9,6.8)	4.2 (3,5.2)	-1.1 (-0.2,-2)
**Costa Rica**	3.3 (2.8,4.1)	5.8 (4.8,6.8)	8.3 (7.1,9.2)	7 (6.1,7.8)	-0.7 (16.2,-15.1)
**Croatia**	6.2 (5.7,6.5)	10.5 (9.6,11.2)	26.4 (23,29.5)	11.6 (10.2,12.9)	-1.5 (-0.7,-2.4)
**Cuba**	18.3 (17.1,19.5)	15.5 (12.8,18.9)	34.7 (30.2,38.9)	18 (15.8,20.1)	-0.5 (-0.1,-0.9)
**Cyprus**	12.9 (12,13.5)	18 (16.7,19.1)	10.9 (8.6,13.2)	9.4 (7.1,11.2)	-2.6 (-1.8,-3.4)
**Czech Republic**	13.8 (11.3,16.9)	15.3 (14.2,16)	18.8 (16.4,21.2)	9.3 (8,10.4)	-0.5 (0,-1)
**Democratic Republic of the Congo**	31.7 (29.5,34.1)	24.2 (19.2,30.1)	7.9 (5.7,10.8)	7.6 (5.6,10.1)	-2.4 (-0.5,-4.3)
**Denmark**	4.7 (3.9,5.7)	26.2 (24.3,28)	3.5 (2.4,5.2)	10.3 (7.2,15.7)	-3.2 (-2.7,-3.7)
**Djibouti**	5.3 (3.8,8)	7.4 (5.4,10.6)	16.9 (14.4,18.4)	7.6 (6.5,8.1)	0.2 (0.5,-0.1)
**Dominica**	3.7 (2.8,4.9)	10.1 (7.7,14.2)	5 (3.3,7.5)	13.9 (9.7,20.2)	-0.1 (0.1,-0.3)
**Dominican Republic**	26 (23.3,27.8)	15.9 (14.3,16.9)	5.1 (3.6,6.4)	4.7 (3.3,5.9)	-2.5 (-1.6,-3.3)
**Ecuador**	3.9 (2.8,5.4)	14 (10.1,18.4)	3 (2.2,4.2)	3.4 (2.5,4.8)	-0.6 (1.4,-2.5)
**Egypt**	3.7 (3.2,4.4)	4.8 (4.2,5.7)	7.3 (6,8.9)	8.5 (7,10.3)	0.4 (10.5,-8.8)
**El Salvador**	2.1 (1.7,2.9)	4.7 (3.7,6.8)	2.4 (1.9,2.9)	4.2 (3.3,5.8)	-0.3 (0.6,-1.2)
**Equatorial Guinea**	5.6 (5.4,5.8)	9.3 (8.9,9.6)	6.2 (4.8,8)	6 (4.6,7.7)	-0.9 (-0.5,-1.2)
**Eritrea**	3 (2.3,3.9)	5.5 (4.3,7.9)	2.5 (1.7,3.7)	8.3 (5.7,12.2)	0 (0.2,-0.3)
**Estonia**	5.8 (4.7,6.5)	8.9 (7.2,10)	5.1 (3.7,7)	16.2 (12,22.3)	-1.3 (-0.6,-1.9)
**Ethiopia**	4.4 (3.1,6.8)	11 (8.2,15.7)	11 (9.7,12.2)	6.1 (5.4,6.8)	-1.6 (0.6,-3.8)
**Fiji**	4.5 (3.4,6.4)	16.1 (12.8,21.4)	5.4 (4.6,6.4)	15.6 (12.8,18.5)	-0.5 (0.2,-1.3)
**Finland**	12.7 (12.1,13.4)	11.2 (10.6,11.7)	4.7 (3.2,6.2)	8.7 (6.3,11.1)	-3.6 (-2.6,-4.6)
**France**	2.1 (1.6,2.6)	5.1 (3.9,6.5)	3.3 (2.5,4.2)	5.8 (4.5,7.1)	-0.2 (1.9,-2.2)
**Gabon**	6.4 (4.1,9.5)	19.9 (13.2,27.6)	26.4 (22.5,28.8)	10.1 (8.9,10.8)	-1.7 (-1.5,-1.9)
**Gambia**	2.5 (2,3.2)	5.9 (4.7,8.2)	27.7 (23.3,30.2)	9.8 (8.5,10.6)	-0.3 (1.9,-2.5)
**Georgia**	18.7 (17.1,19.8)	14.1 (12.8,14.9)	4.3 (3.2,6.2)	9.8 (7.1,14.2)	-1.8 (-1.1,-2.5)
**Germany**	23 (20.7,24.4)	15.8 (14.2,16.7)	12.2 (11,13.5)	8.4 (7.6,9.3)	-1.5 (-1.2,-1.7)
**Ghana**	6 (4.6,8.1)	12.2 (9.3,16.5)	26.3 (22,28.8)	9.4 (8.1,10.2)	-1.1 (0.3,-2.6)
**Greece**	5.9 (4.7,7.2)	17.6 (14,21.6)	5.8 (4.4,7.3)	15 (11.8,18.8)	-0.4 (0.9,-1.7)
**Greenland**	8.1 (7.6,8.6)	8 (7.5,8.5)	13.9 (11.9,15)	4.6 (4.1,4.9)	1.3 (1.9,0.8)
**Grenada**	16.2 (14.5,17.3)	10.5 (9.5,11.2)	15.2 (10.1,19.2)	17.2 (11.3,22.2)	-1.5 (-1.2,-1.8)
**Guam**	5.5 (4.4,6.7)	15.4 (11.8,19.2)	6.9 (6.1,7.6)	7.5 (6.8,8.3)	-0.1 (0.2,-0.5)
**Guatemala**	5.8 (5.3,6.1)	4.6 (4.2,4.8)	3.1 (2.5,3.6)	2.4 (2,2.8)	-1 (-0.2,-1.8)
**Guinea**	12.4 (8.7,15.1)	25.1 (17.5,30.6)	5.9 (5.1,6.8)	8.3 (7.2,9.4)	-1.3 (3.7,-6.1)
**Guinea-Bissau**	5.8 (5.3,6.4)	6.1 (5.6,6.6)	5.6 (4.2,7.2)	13.8 (10.6,17.8)	0.6 (7.2,-5.6)
**Guyana**	2.6 (2.2,3.3)	6.4 (5.2,8.1)	4.9 (3.8,6.6)	16.2 (12.6,21.7)	-0.4 (5.1,-5.6)
**Haiti**	7.2 (6.9,7.4)	15.5 (14.8,16.1)	8.4 (6.7,10.2)	12.1 (9.8,14.5)	-1.8 (-1.4,-2.1)
**Honduras**	7.7 (5.9,9.8)	14.4 (10.8,18.8)	3.7 (2.5,5)	8.6 (6.1,11.3)	-0.2 (0.4,-0.8)

**Hungary**	6.8 (4.9,9.7)	18.3 (14,24.4)	3.3 (2.5,4.2)	5.5 (4.3,7)	-0.5 (0.7,-1.8)
**Iceland**	5.9 (5.3,6.5)	12 (10.8,13.2)	18.7 (16.3,21.1)	9.1 (8,10.2)	0.2 (1.8,-1.4)
**India**	4 (2.8,5.3)	9.3 (7.3,11.4)	15 (12.2,16.8)	7.8 (6.5,8.7)	-0.5 (0.2,-1.1)
**Indonesia**	2.9 (2.4,3.5)	5.6 (4.5,7.3)	17 (13.9,19.2)	24.2 (19.7,27.3)	-1.1 (-0.4,-1.8)
**Iran**	39.3 (36.5,41.7)	33.3 (30.9,35.4)	7 (5.6,8.5)	10.8 (8.3,12.7)	-4.5 (-4,-4.9)
**Iraq**	8.2 (7.3,8.8)	6.9 (6.2,7.4)	2 (1.6,2.6)	3.3 (2.6,4.3)	0.1 (4.2,-3.8)
**Ireland**	12.3 (9.2,14.3)	26.7 (20.4,31.5)	7.1 (5.9,7.7)	4.3 (3.6,4.6)	-0.8 (-0.5,-1)
**Israel**	5.9 (4.6,6.9)	11.5 (8.3,14.1)	3.2 (2.7,4.2)	3.8 (3.2,5.2)	-0.9 (-0.8,-1)
**Italy**	3.7 (3.1,4.3)	6 (5.2,7.5)	5.4 (4.4,5.9)	3.7 (3.1,4)	-1.7 (-1.4,-1.9)
**Ivory Coast**	2.9 (2.3,3.6)	4.4 (3.6,5.4)	20.8 (16.7,22.9)	6.4 (5.3,6.9)	-1.7 (-1.3,-2.1)
**Jamaica**	7.8 (7.2,8.2)	7.8 (7.1,8.2)	3.9 (3.1,4.8)	3.1 (2.4,3.8)	-1.9 (-0.8,-3.1)
**Japan**	4.4 (4.1,4.7)	5.3 (4.8,5.6)	12.8 (10.1,14.2)	3.3 (2.8,3.5)	-1.2 (-0.2,-2.3)
**Jordan**	15.3 (13.5,16.3)	10.9 (9.6,11.6)	1.9 (1.5,2.3)	3.3 (2.6,4.2)	-2.5 (-2.2,-2.8)
**Kazakhstan**	1.8 (1.6,1.9)	2.2 (2,2.3)	3.9 (3.5,4.3)	4.1 (3.7,4.5)	0.3 (2.5,-2)
**Kenya**	4 (3.7,4.2)	3.4 (3.1,3.5)	5.8 (4.4,7.2)	16.8 (13,20.7)	-2 (-1.8,-2.3)
**Kiribati**	2.9 (2.4,3.4)	5.8 (4.9,6.9)	2.3 (1.5,3.5)	4.2 (2.9,6.5)	-2.4 (-1.6,-3.3)
**Kuwait**	6.3 (5.8,6.7)	6.7 (6.3,7.2)	1.2 (0.7,1.6)	2.8 (1.7,3.6)	-1 (-0.7,-1.4)
**Kyrgyzstan**	5.3 (3.6,6.8)	16.8 (11.5,21.6)	3.1 (2.6,3.8)	4.8 (4,5.7)	-0.1 (0.2,-0.4)
**Laos**	1.2 (0.8,1.5)	2.7 (1.8,3.4)	2.9 (2.5,3.4)	3.4 (2.9,3.9)	-0.2 (10.7,-10.1)
**Latvia**	2.7 (2.5,2.8)	5.1 (4.6,5.5)	6.1 (4.6,7.9)	10 (7.8,12.5)	-1.4 (-0.2,-2.6)
**Lebanon**	6.2 (5.8,6.6)	6.9 (6.5,7.4)	14.8 (13.2,16.3)	8.1 (7.2,8.9)	-2.7 (-2,-3.3)
**Lesotho**	6 (4,9.3)	11.1 (8,15.5)	7.3 (6.1,8.6)	6.1 (5.1,7.2)	-0.8 (-0.2,-1.3)
**Liberia**	16.9 (16,17.7)	14.5 (13.7,15.1)	2.9 (2,4.4)	4.9 (3.3,7.5)	-3.9 (-3.2,-4.6)
**Libya**	6 (4.4,7.5)	10.4 (6.8,13.3)	4.6 (3.4,6)	13.2 (10.1,17.1)	-0.7 (0.1,-1.6)
**Lithuania**	2.5 (1.7,3.6)	4.6 (3.1,6.6)	5.3 (3.8,7.9)	7 (4.7,11.5)	0.9 (2.4,-0.6)
**Luxembourg**	6.7 (5.5,8.2)	15.1 (12.5,18.6)	17.9 (16,19.7)	9.5 (8.6,10.4)	-0.3 (0.7,-1.3)
**Madagascar**	3.8 (2.9,5.1)	6.4 (4.7,9.5)	15.8 (13.3,17.6)	8.4 (7.2,9.3)	0.7 (1.4,0)
**Malawi**	13.1 (12.3,13.8)	11.7 (11.1,12.4)	3.1 (2.3,4.3)	10 (7.4,13.9)	-2.3 (-1.7,-2.9)
**Malaysia**	13.7 (12.7,14.5)	10.8 (10,11.3)	5.8 (4.4,7.2)	17.8 (14,22.1)	-2.4 (0.4,-5.1)
**Maldives**	4.8 (3.8,5.9)	12.6 (10.1,15.6)	5 (3.7,5.8)	6.2 (4.5,7.2)	-0.8 (-0.3,-1.2)
**Mali**	6.9 (5.5,8)	18.8 (15.1,22.2)	3.4 (2.6,5)	4.9 (3.9,6)	-0.5 (-0.1,-1)
**Malta**	4.3 (3.3,4.8)	7.4 (5.7,8.6)	5.2 (3.6,6.8)	15.9 (11.2,20.9)	-1.3 (-1,-1.6)
**Marshall Islands**	3.7 (2.3,5.4)	8.1 (5.4,10.9)	14.7 (12.1,16.4)	6.2 (5.3,6.9)	-1.8 (1.4,-4.8)
**Mauritania**	7.1 (5.7,9)	18 (14.3,23.2)	3.9 (2.5,5.5)	8.2 (5.5,10.8)	-0.4 (0.1,-0.9)
**Mauritius**	8.6 (7.8,9.2)	8.6 (7.7,9.2)	5.1 (3.6,6.7)	11.8 (8.7,15.4)	-0.4 (2.9,-3.7)
**Mexico**	3 (2.4,3.6)	8.6 (7,10.2)	5.2 (4.8,5.5)	4 (3.7,4.2)	0.1 (7.9,-7.2)
**Micronesia**	6.2 (5.1,7.5)	14.2 (11.8,17.5)	5.7 (5,6.3)	6.2 (5.5,6.8)	-1 (0.1,-2.1)
**Moldova**	1.7 (1.6,1.8)	2.4 (2.2,2.5)	5.5 (4.5,6.4)	6.3 (5.1,7.4)	0.7 (2.4,-1.1)
**Monaco**	7.5 (7.4,7.7)	14.4 (13.8,14.7)	8.9 (7.4,10.7)	6.9 (5.7,8.3)	-2.4 (-2.3,-2.6)
**Mongolia**	4 (3.3,5)	9.2 (7.4,11.2)	4.5 (3.5,6.3)	5.5 (4.3,7.8)	-0.2 (5.1,-5.2)
**Montenegro**	9.2 (6.7,11.6)	3.8 (2.9,4.7)	4.9 (2.6,7)	15.9 (8.9,22.2)	-0.4 (16,-14.5)
**Morocco**	7.3 (5.2,9)	8.8 (6.8,10.8)	10.7 (8.3,14.6)	14 (10.8,18.2)	-1 (-0.2,-1.8)

**Mozambique**	7.2 (5.9,8.6)	7.6 (6.2,9)	2.4 (1.7,3.3)	4.2 (3.1,5.8)	-1.2 (0.8,-3.2)
**Myanmar**	4.9 (3.6,6.3)	6.9 (5.1,9.3)	10.1 (7.8,14.3)	16.1 (12.1,24.1)	-0.9 (-0.7,-1.2)
**Namibia**	6.1 (3.6,8.1)	15.7 (9.5,20.6)	33.3 (27.8,36.5)	14.2 (12,15.5)	0.6 (1,0.2)
**Nauru**	10.1 (7.3,13.4)	17.3 (13.5,22)	13.3 (11.1,14.5)	7.4 (6.3,8)	-1.3 (-1.1,-1.4)
**Nepal**	2.1 (1.6,2.6)	4.7 (3.7,5.9)	3.4 (2.8,4.1)	4.9 (4.1,5.8)	-0.5 (1,-2)
**Netherlands**	3.5 (2.5,4.8)	8.5 (6.7,10.6)	4.5 (2.9,6.2)	14.7 (9.6,20.7)	-0.1 (16.9,-14.7)
**New Zealand**	8.7 (6.2,12.2)	17 (12.5,23.7)	4.3 (3.5,5.2)	12.2 (10.1,14.8)	-0.6 (-0.4,-0.9)
**Nicaragua**	11.4 (10,12.3)	8.8 (7.7,9.4)	5.3 (4.4,6.1)	7.7 (6.2,8.9)	-0.3 (0.2,-0.9)
**Niger**	7.1 (6.4,7.6)	6.9 (6.2,7.4)	25.8 (21.4,28.2)	11.2 (9.4,12.2)	-0.1 (1.1,-1.2)
**Nigeria**	3.5 (2.8,3.9)	7 (5.6,8)	2.9 (2.3,3.5)	6 (4.6,7.2)	-1.4 (-0.6,-2.3)
**Niue**	5.7 (4.3,7.3)	15.8 (11.9,21)	5.6 (3.5,7.6)	12.1 (7.8,16.4)	-0.6 (-0.1,-1.1)
**North Korea**	6.1 (4.9,7.6)	13.7 (11.1,16.9)	2.8 (2.3,3.3)	5.6 (4.7,6.5)	-0.9 (-0.7,-1.1)
**North Macedonia**	8.3 (6.6,10.6)	7.8 (6.2,10)	2.8 (2.3,3.4)	2.7 (2.2,3.2)	-0.2 (50,-33.7)
**Northern Mariana Islands**	4.4 (3.8,5.1)	5.1 (4.4,5.9)	4.1 (2.3,6.1)	8.7 (4.6,13.5)	-1.5 (-0.1,-2.9)
**Norway**	2.8 (2.1,3.8)	8.2 (6.6,10.8)	4.4 (2.8,5.8)	5.6 (3.6,7.5)	-0.3 (8.7,-8.5)
**Oman**	21.3 (19,22.7)	12.2 (10.9,12.9)	5 (3.4,6.5)	5.3 (3.6,6.8)	-1.7 (-0.6,-2.8)
**Pakistan**	4.7 (3.5,6.3)	9.3 (6.8,12.3)	4.4 (3.5,5.2)	6.4 (5,7.4)	-1.6 (-0.7,-2.5)
**Palau**	6.8 (4.2,9.1)	13.7 (8.5,19.1)	6.2 (4.4,8.5)	8.3 (5.9,11.4)	-0.7 (-0.6,-0.8)
**Palestine**	9.8 (7.7,12.6)	19.1 (14.9,24.9)	15 (13.4,16.3)	8.3 (7.6,9)	-0.2 (8.8,-8.5)
**Panama**	4.7 (3.7,5.7)	8.9 (7.2,11)	15 (12.9,16.3)	5.5 (4.8,5.9)	-1.7 (-0.6,-2.7)
**Papua New Guinea**	3.8 (3.5,3.9)	5.7 (5.3,6)	11.2 (7.8,14)	3.8 (2.7,4.7)	-1.9 (-0.7,-3)
**Paraguay**	3.5 (2.3,4.9)	8.2 (5,12)	11 (9,12.8)	4.3 (3.6,5.1)	0.3 (0.9,-0.4)
**Peru**	2.6 (2.2,3.3)	4.5 (3.7,5.7)	3.4 (2.4,4.7)	7.3 (5.6,9.5)	0.3 (1.3,-0.6)
**Philippines**	3.8 (3.2,4.7)	5.9 (4.9,7.1)	4.7 (3.3,6.5)	6 (4.3,8.5)	-0.6 (-0.3,-0.9)
**Poland**	3.5 (3.1,4.1)	6.8 (5.7,8.5)	4.7 (3.7,6.1)	13.5 (11,16.9)	-0.2 (0,-0.4)
**Portugal**	12.8 (12.2,13.2)	12.5 (11.8,12.9)	9.9 (7.5,12.2)	6 (4.6,7.4)	-1.3 (-1,-1.6)
**Puerto Rico**	8.2 (7.7,8.6)	7.2 (6.8,7.6)	7.3 (6.5,8.1)	5 (4.5,5.7)	-2.3 (-1.7,-3)
**Qatar**	5.2 (4.9,5.4)	5.9 (5.5,6.2)	3.9 (2.3,6.1)	8.8 (5.5,12.9)	-0.8 (0.5,-2.1)
**Republic of Congo**	5.4 (4.2,6.6)	15.8 (11.2,19.5)	8.7 (6.1,10.7)	7.9 (5.6,9.8)	-2 (-0.5,-3.5)
**Romania**	7.2 (5.7,8.9)	10 (8.6,14.1)	12.7 (10.1,15.6)	16.9 (13.6,20.6)	-3.6 (-3.4,-3.9)
**Russia**	8 (7.6,8.5)	8.4 (8,8.9)	7.9 (5.1,10.9)	2.5 (1.7,3.4)	-2.1 (-1.5,-2.7)
**Rwanda**	10.4 (9.8,11.1)	10.3 (9.8,10.9)	6.5 (4.9,8)	17.6 (13.1,21.8)	-2.6 (-2.3,-2.9)
**Saint Kitts**	6.1 (6,6.2)	5.9 (5.8,6)	10.8 (9.5,12)	6.3 (5.5,6.9)	-1.4 (-1.1,-1.6)
**Saint Lucia**	6.4 (4.9,8.5)	19 (15.1,24.9)	8.6 (8,9.3)	6.1 (5.7,6.6)	-1.9 (-1.4,-2.4)
**Saint Vincent**	5.7 (5.4,6.1)	7.2 (6.7,7.5)	5.4 (3.7,8.1)	14.7 (10.7,21.3)	-0.5 (9.7,-9.8)
**Samoa**	2.6 (2.5,2.8)	5 (4.7,5.3)	6.2 (5.4,6.9)	7.2 (6.4,8)	-0.2 (6.3,-6.3)
**San Marino**	3.9 (3.6,4.2)	6.1 (5.7,6.6)	5.7 (4.7,6.7)	4.6 (3.8,5.4)	0.6 (6.2,-4.7)
**Sao Tome and Principe**	3.7 (2.8,4.8)	8.1 (6.1,10.9)	9.1 (8.2,10.1)	8.5 (7.7,9.4)	0 (4.4,-4.3)
**Saudi Arabia**	7.5 (6.1,9.3)	5 (4,6.2)	4.5 (3.1,6)	7.8 (5.4,10)	-0.9 (17.2,-16.2)
**Senegal**	8.7 (7.3,10.4)	14.8 (12.4,18.5)	6.8 (5.4,9)	14.3 (11.4,18.6)	-0.4 (3.3,-3.9)
**Serbia**	3.7 (2.6,5.2)	7 (4.8,10)	2.8 (1.8,4.4)	5 (3.4,7.1)	-1.4 (-1,-1.7)
**Seychelles**	6.4 (5.3,7.8)	15.3 (12.5,18.8)	6.3 (4.4,8.3)	14.5 (10.6,18.8)	-0.2 (0.3,-0.7)

**Sierra Leone**	6.6 (5.4,7.7)	7.4 (6.1,8.6)	7.8 (5.9,9.3)	4.4 (3.4,5.2)	-2.1 (-1.5,-2.8)
**Singapore**	6.1 (4.6,7.2)	7.5 (5.7,8.9)	4 (3.3,5)	3.8 (3.2,4.8)	-1.6 (4,-6.9)
**Slovakia**	6.9 (5.6,8.5)	14.2 (11.5,17.6)	5.1 (3.7,6.5)	12.8 (9.8,16.3)	-0.1 (0.7,-0.8)
**Slovenia**	3.3 (3.2,3.5)	4.4 (4.2,4.6)	3.7 (3.3,3.9)	2.7 (2.4,2.9)	-3.2 (-2.2,-4.2)
**Solomon Islands**	15.9 (11.1,18.9)	15.2 (10.8,18)	14.7 (11.8,17.7)	9.2 (7.4,11)	-1.5 (-0.9,-2.1)
**Somalia**	21 (19.6,22.2)	17.9 (16.7,18.8)	32 (27.1,35.5)	12.4 (10.6,13.7)	-3.7 (-2.7,-4.8)
**South Africa**	3.4 (2.1,4.5)	8.8 (6,11.4)	15 (9.5,19.4)	18.3 (10.5,24.2)	0.2 (2.6,-2.1)
**South Korea**	4.5 (3,6.2)	16.7 (11.8,23.2)	4.3 (2.8,5.8)	9.4 (6.2,13)	-0.3 (0.1,-0.8)
**South Sudan**	2 (1.6,2.3)	3.2 (2.4,3.9)	3.7 (2.4,5.5)	14.9 (10.1,22.3)	-1 (-0.6,-1.3)
**Spain**	5.7 (4,7.7)	14.7 (10.4,19.8)	1.9 (1.7,2.4)	2.5 (2.2,3.1)	-0.3 (0.4,-0.9)
**Sri Lanka**	5.8 (5.5,6.1)	4.7 (4.4,4.9)	5.1 (3.6,7.2)	14.5 (10.1,20.7)	-2 (-1.7,-2.3)
**Sudan**	8.5 (7,9.7)	13.6 (11.4,15.5)	10.5 (8.9,11.6)	4.1 (3.6,4.5)	-2.5 (-2.2,-2.7)
**Suriname**	5.5 (3,8.8)	8 (5,11.4)	8 (5.4,11.2)	7.5 (5.2,10.4)	-1.3 (-1,-1.6)
**Swaziland**	3.9 (3.2,5.6)	6.1 (5,9.1)	3.3 (2.4,4.5)	5.5 (4,7.9)	-0.3 (2.8,-3.3)
**Sweden**	14.9 (13.2,15.9)	7.9 (7.1,8.4)	5.4 (4,7.1)	5.5 (4,7.3)	-0.8 (0.2,-1.8)
**Switzerland**	26 (23.1,27.8)	16.4 (14.7,17.4)	19.9 (16.8,22.4)	7.5 (6.4,8.4)	-2.3 (-1.7,-3)
**Syria**	2.5 (1.9,3.1)	4.6 (3.8,5.5)	24.4 (19.7,27.2)	9.3 (7.7,10.2)	-1.6 (-1,-2.2)
**Taiwan**	5.8 (5.6,6)	7.3 (7,7.7)	2.3 (1.8,3)	3.4 (2.7,4.2)	-1.7 (-1.3,-2)
**Tajikistan**	5.7 (4.2,6.9)	6.4 (5,7.4)	10.1 (9.1,11)	5.9 (5.4,6.3)	-1.3 (-0.7,-1.9)
**Tanzania**	5.4 (4.4,6.5)	8.9 (7.2,10.7)	4.5 (2.8,6.4)	4.8 (3.2,6.5)	-0.6 (-0.4,-0.7)
**Thailand**	4.4 (3.3,6.1)	9.2 (6.3,12.9)	9.5 (7.1,13)	6.5 (4.9,8.6)	-0.3 (1.3,-1.8)
**Timor-Leste**	4.8 (4,5.8)	14.4 (12,17.8)	5.5 (4,6.9)	5.2 (3.9,6.5)	-0.4 (0.3,-1.2)
**Togo**	6.1 (4.5,7.8)	8.2 (6.1,10.6)	5.7 (4.1,7.5)	9.6 (6.8,12.5)	-0.2 (61.4,-38.3)
**Tokelau**	2.4 (2,3.1)	5.3 (4.1,7.3)	4.9 (3.7,6.3)	13.5 (10.7,17.2)	-0.4 (8.6,-8.6)
**Tonga**	3.8 (3.6,4)	6.9 (6.5,7.3)	8.5 (5.9,11)	8.3 (5.8,10.8)	-0.7 (1.1,-2.5)
**Trinidad**	4.1 (3.3,5.4)	6.3 (4.9,9.1)	3.4 (2.6,4.4)	4.8 (3.6,6.2)	-1.4 (-0.9,-2)
**Tunisia**	3.7 (3.5,3.9)	10.2 (7.9,12.5)	5.9 (4.7,7.3)	4.7 (3.7,5.8)	-1.1 (-0.8,-1.3)
**Turkey**	5 (4.2,6)	4.1 (4,4.3)	4.3 (3,6.8)	4.5 (3.1,7.2)	-1 (-0.1,-2)
**Turkmenistan**	6.1 (4.7,7.8)	9.5 (7.6,11.6)	6.8 (5.3,8.1)	7.1 (5.5,8.6)	-0.1 (16.3,-14.2)
**Tuvalu**	4.3 (3.3,5.6)	13.4 (9.8,18.1)	2.3 (1.9,2.8)	2.5 (2.1,3)	-0.8 (-0.4,-1.1)
**Uganda**	7.8 (7.4,8.1)	6.9 (6.7,7.2)	5.9 (4.2,7.7)	8.8 (6.1,11.5)	-3.6 (-3.4,-3.8)
**UK**	5.1 (4,6.5)	11.4 (8.4,14.6)	3.7 (2.8,4.9)	12.1 (9.1,17.2)	-1.8 (-1,-2.6)
**Ukraine**	7.8 (7.1,8.1)	5.1 (4.7,5.3)	6.3 (4.6,8.1)	4.4 (3.2,5.6)	0 (0.3,-0.3)
**United Arab Emirates**	6.5 (5.5,7.6)	16.7 (13.9,20.6)	2.7 (2,3.6)	7.9 (6.1,9.9)	-0.9 (-0.6,-1.1)
**Uruguay**	5.4 (4.8,5.7)	4.3 (3.8,4.5)	15.3 (13.2,16.3)	6.9 (6.1,7.4)	0.7 (0.8,0.5)
**USA**	3.7 (3.1,4.4)	6.2 (5.2,7.4)	5.5 (4.2,7.1)	13.9 (11.2,18.3)	-0.6 (6.6,-7.3)
**Uzbekistan**	6.7 (6.3,7.1)	5.8 (5.5,6.2)	15.2 (13,16.4)	8.1 (7,8.6)	-0.9 (0.3,-2)
**Vanuatu**	4.3 (4.1,4.6)	4.7 (4.5,5)	6.1 (4.5,7.8)	3.8 (2.9,4.8)	-1.9 (-1.6,-2.3)
**Venezuela**	3.1 (2.3,3.9)	8.1 (6,10.2)	10.1 (8.8,10.9)	5.3 (4.8,5.7)	0 (3.7,-3.6)
**Vietnam**	4 (3.8,4.1)	7.2 (6.7,7.5)	2.2 (1.9,2.5)	2.4 (2.2,2.8)	-1.9 (-1.5,-2.3)
**Virgin Islands**	10.5 (7.2,13.8)	18.8 (11.7,25.9)	4 (2.8,5)	8.5 (6,10.8)	-0.4 (-0.3,-0.6)
**Yemen**	4.5 (2.8,6.8)	8 (5.4,10.9)	3.3 (2.5,4.2)	6.4 (4.5,8.9)	-1.2 (-0.8,-1.6)

**Zambia**	6.7 (5.5,8)	20 (16.2,23.7)	5.2 (3.9,6.6)	16.6 (13.2,20.9)	-0.8 (-0.4,-1.3)
**Zimbabwe**	2.4 (1.9,3.2)	6.6 (5.2,8.3)	2.8 (1.8,4.4)	6.5 (4.3,9.7)	0.7 (1.3,0.1)
ASMR=Age-standardised mortality rate.<br>UI: Uncertainty interval;CI: Confidence interval.					

Figure 1S. Globally, the elderly rank of unintentional injury deaths by all causes, 1990 VS 2021.

Figure2S. The proportion of causes of unintentional injury deaths in the whole population globally and in different SDI regions in 2021.

(A) The number and proportion of unintentional injury death among different mental disease in 2021, by SDI quintiles.

The proportion of the mortality rate from falls in the whole population due to intentional injuries in 1990 and 2021,by SDI quintiles.

Figure 3S. The Local drifts of falls mortality (estimates from APC models) for 18 age groups (＜5 to 85+ years) by SDI quintiles, 1990−2021.

Figure 4S. Age-Period-Cohort Modeling: Estimation of mortality of falls by SDI quintiles from 1990 to 2021.

Figure 5S. Gender-specifc burden in terms of age-standardized mortality rates (ASMR) of falls by SDI quintiles, 1990-2021.

Figure 6S. Decomposition analysis of changes in falls deaths according to population-level determinants of population growth, aging, and epidemiological change from 1990 to 2021 by SDI quintiles(A) and gender(B). Black dots represent the overall value of change contributed by all three components.

Figure 7S. Global falls incidence and deaths trends prediction by gender. Globally, the number and age standardized rate (ASIR and ASMR) (a, b) of incidence and deaths trends of falls for all age groups (0-14 to 95+ years) from 1992 to 2021 and projections from 2022 to 2046 by gender.

Figure 8S. Global the elderly with falls deaths trends prediction by gender. The number and age standardized rate (ASMR) of falls for the elderly (65-69 to 95+ years) from 1992 to 2021 and projections from 2022 to 2046 by gender.

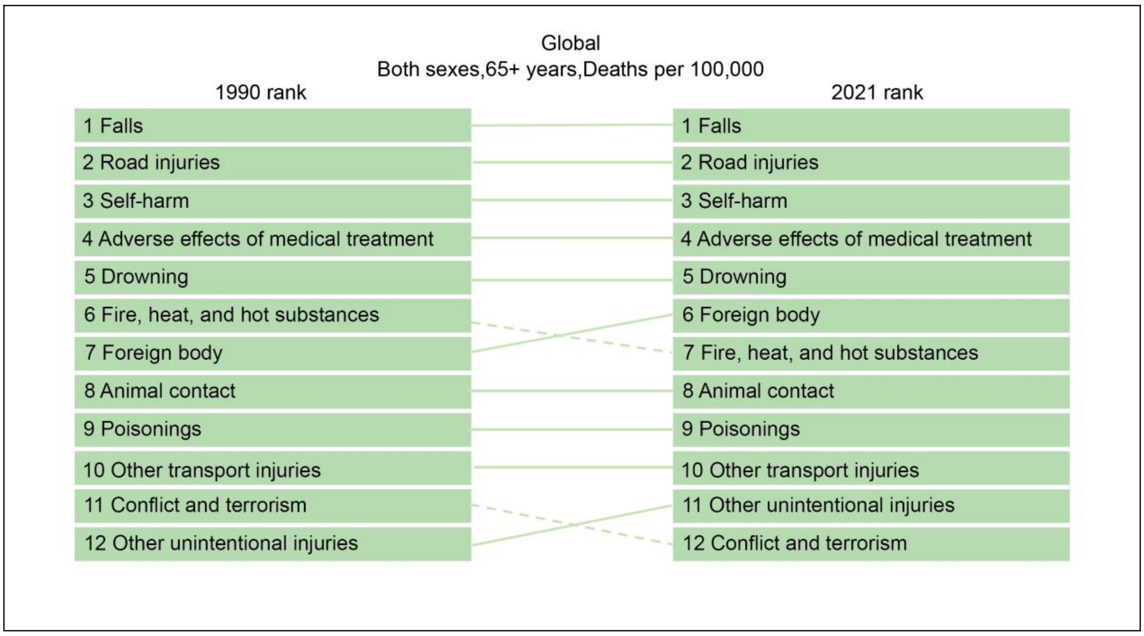

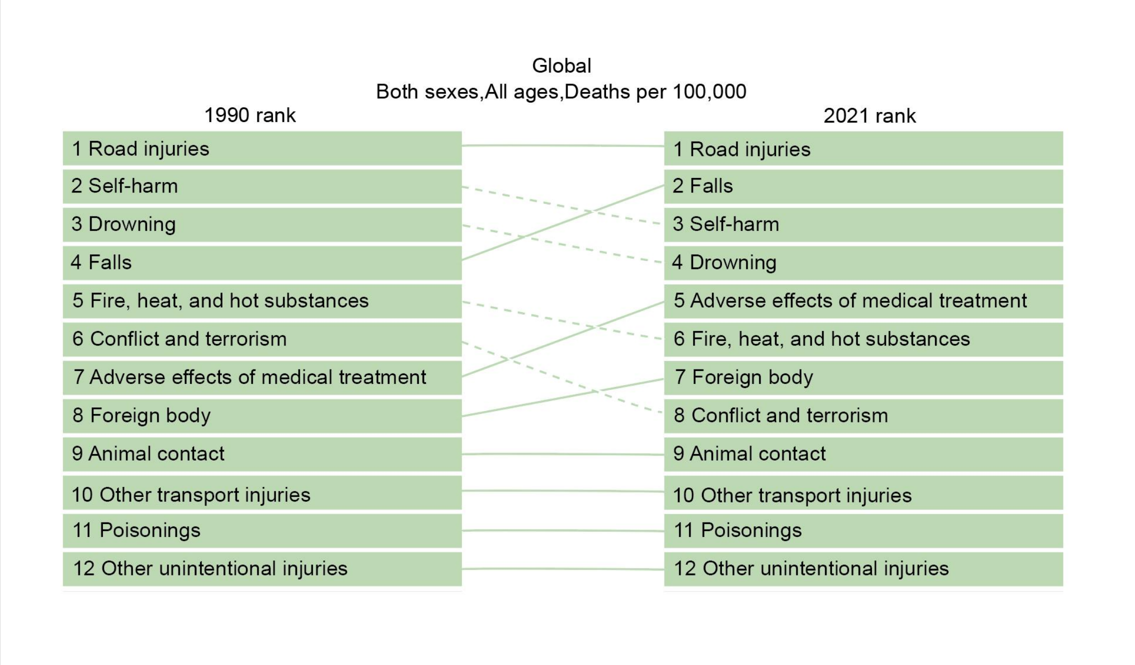
Global
Both sexes,All ages,Deaths per 100,000
1990 rank
1 Road injuries
2 Self-harm
3 Drowning
4 Falls
5 Fire, heat, and hot substances
6 Conflict and terrorism
7 Adverse effects of medical treatment
8 Foreign body
9 Animal contact
10 Other transport injuries
11 Poisonings
12 Other unintentional injuries
2021 rank
1 Road injuries
2 Falls
3 Self-harm
4 Drowning
5 Adverse effects of medical treatment
6 Fire, heat, and hot substances
7 Foreign body
8 Conflict and terrorism
9 Animal contact
10 Other transport injuries
11 Poisonings
12 Other unintentional injuries

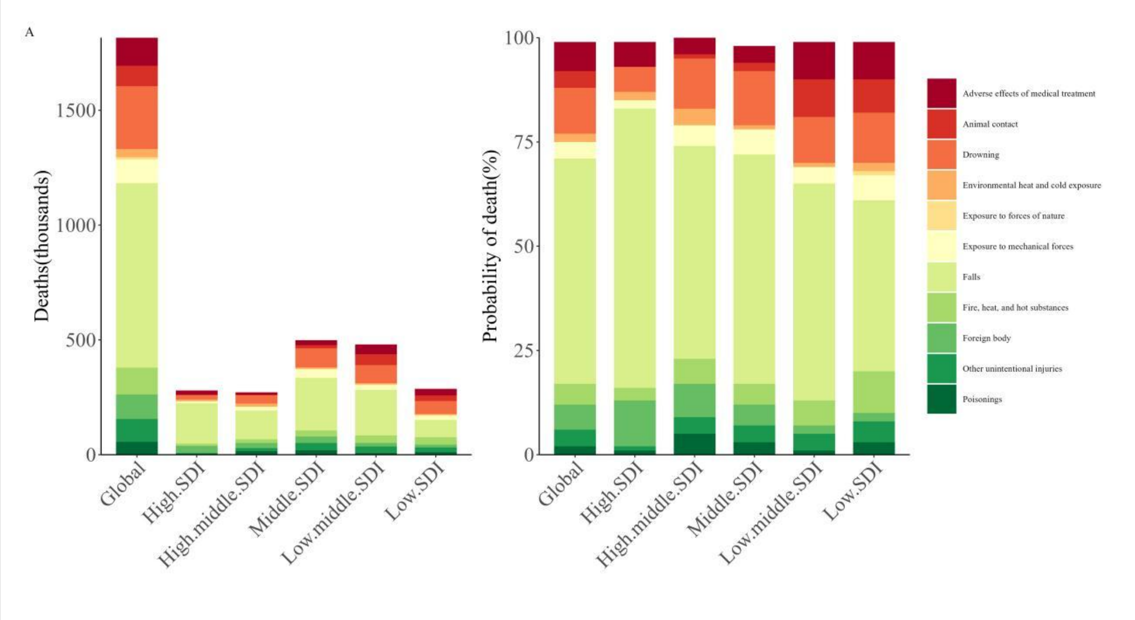
A
Deaths(thousands)
1500
1000
500
0
Global
High.SDI
High.middle.SDI
Middle.SDI
Low.middle.SDI
Low.SDI
Probability of death(%)
100
75
50
25
0
Adverse effects of medical treatment
Animal contact
Drowning
Environmental heat and cold exposure
Exposure to forces of nature
Exposure to mechanical forces
Falls
Fire, heat, and hot substances
Foreign body
Other unintentional injuries
Poisonings

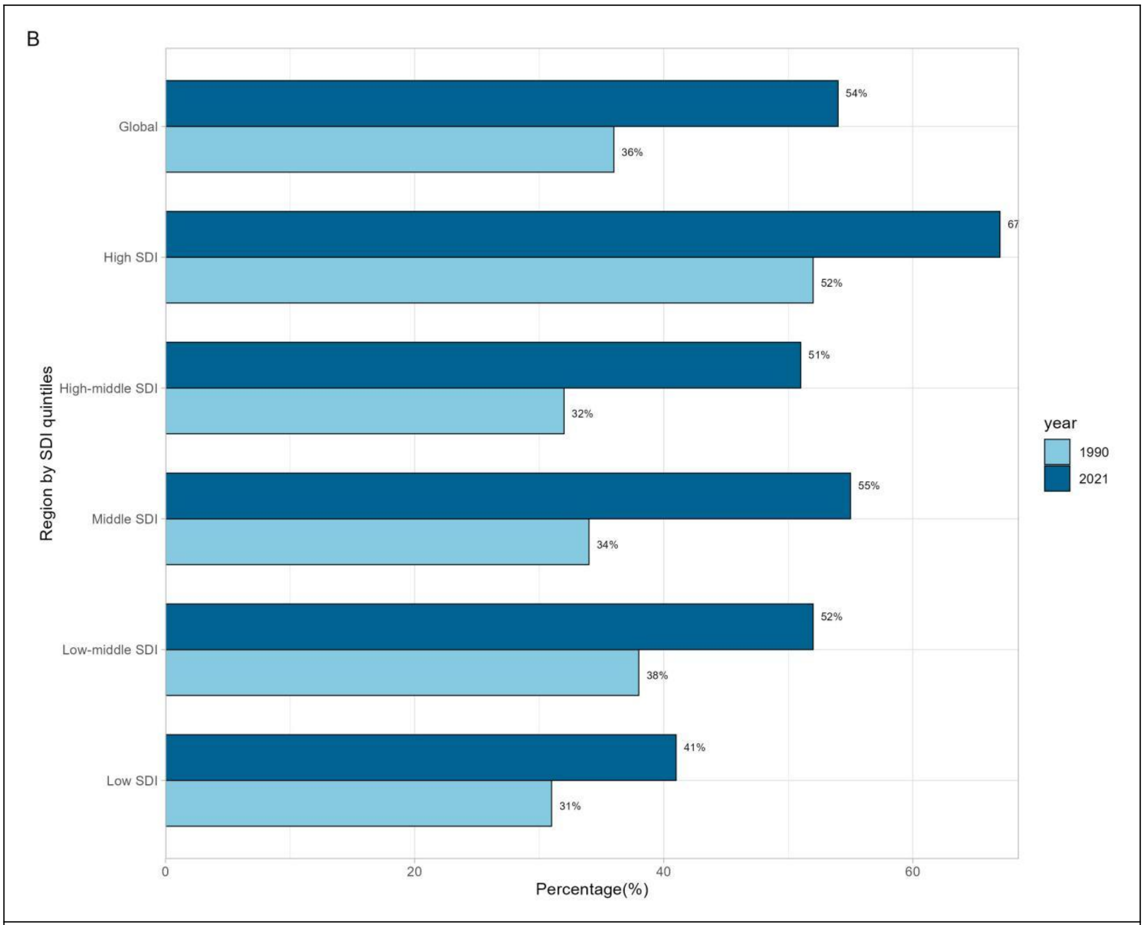

B
Global
High SDI
High-middle SDI
Middle SDI
Low-middle SDI
Low SDI
Region by SDI quintiles
54%
36%
67
52%
51%
32%
55%
34%
52%
38%
41%
31%
0
20
40
60
Percentage(%)
year
1990
2021

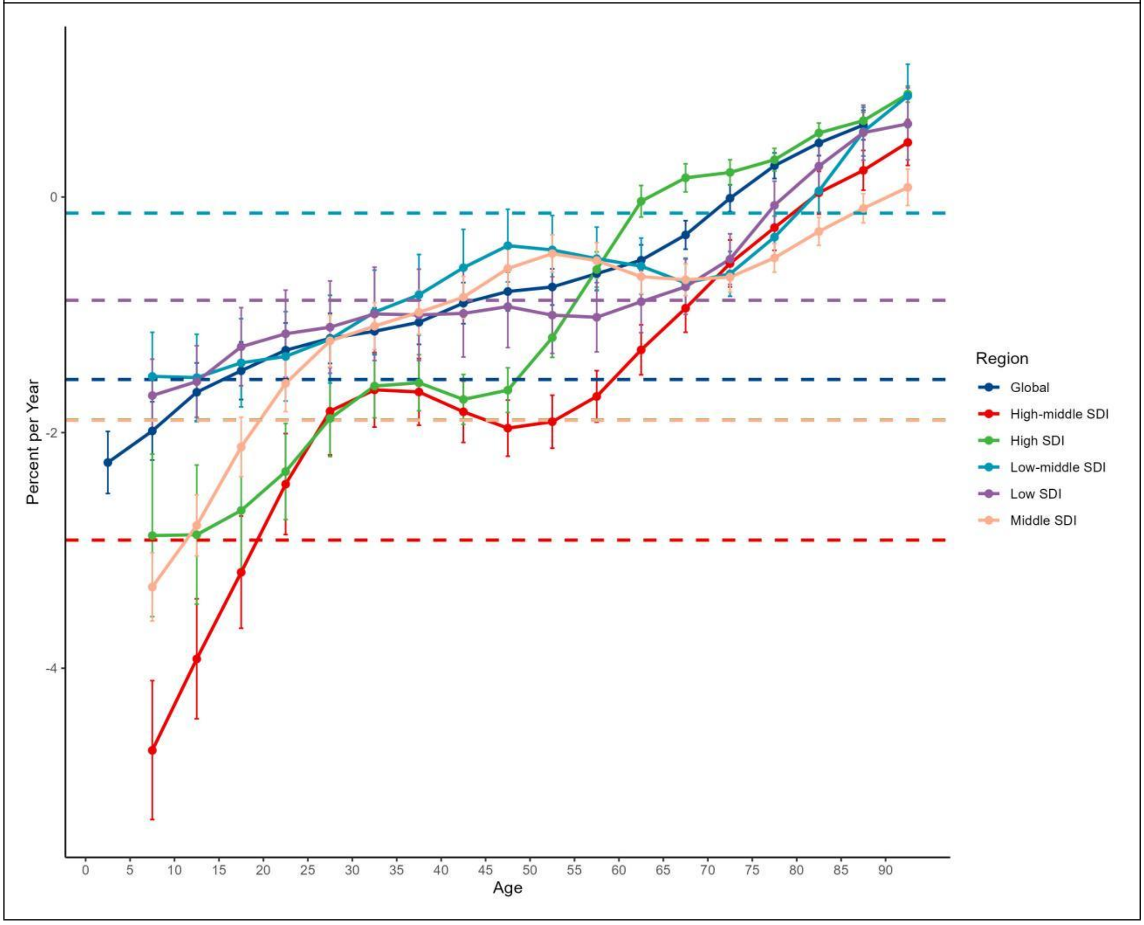

Percent per Year
0
-2
-4
Age
0 5 10 15 20 25 30 35 40 45 50 55 60 65 70 75 80 85 90
Region
Global
High-middle SDI
High SDI
Low-middle SDI
Low SDI
Middle SDI

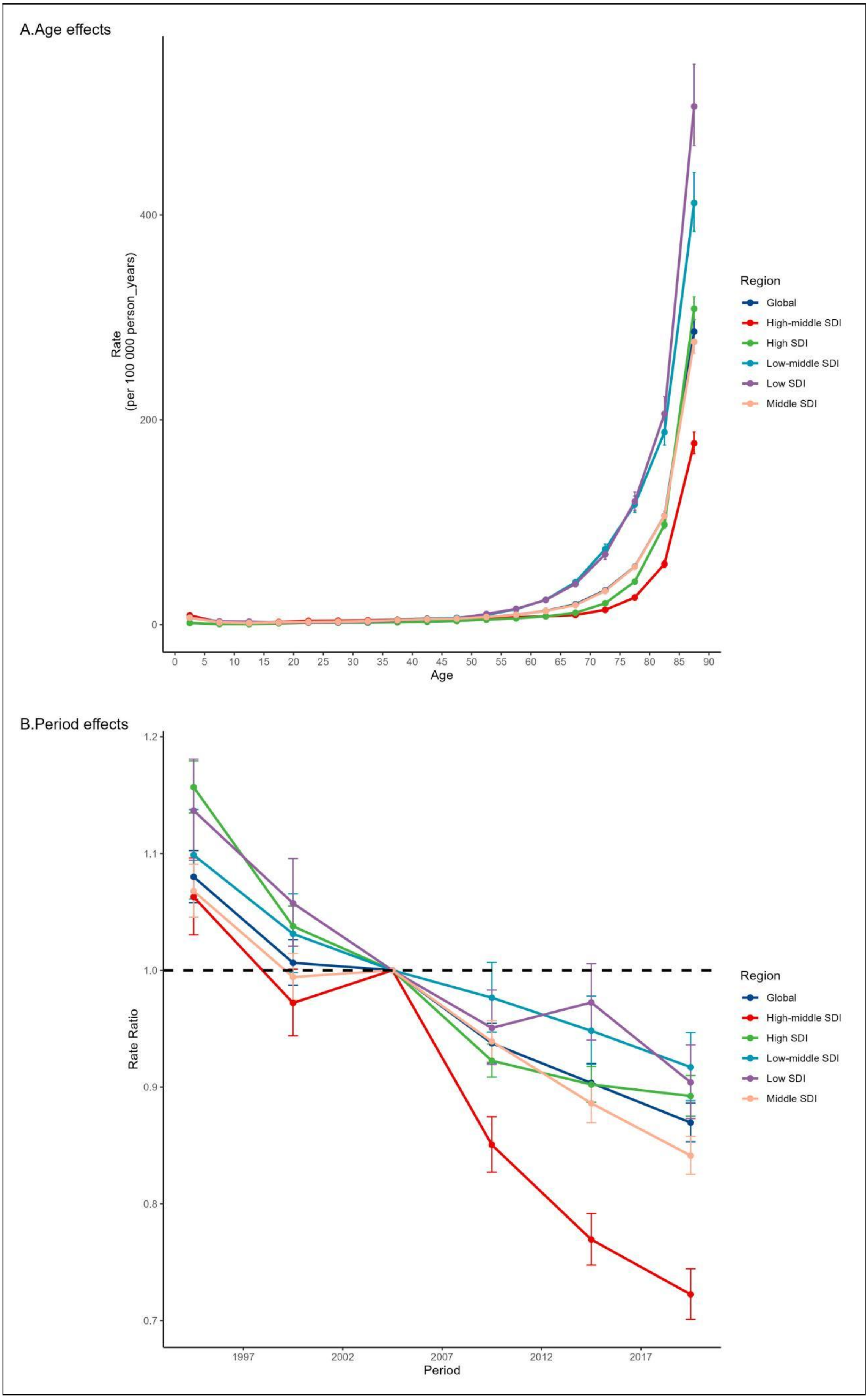
A.Age effects
Rate
(per 100 000 person_years)
400
200
0
0 5 10 15 20 25 30 35 40 45 50 55 60 65 70 75 80 85 90
Age
Region
Global
High-middle SDI
High SDI
Low-middle SDI
Low SDI
Middle SDI
B.Period effects
Rate Ratio
1.2
1.1
1.0
0.9
0.8
0.7
1997
2002
2007
2012
2017
Period
Region
Global
High-middle SDI
High SDI
Low-middle SDI
Low SDI
Middle SDI

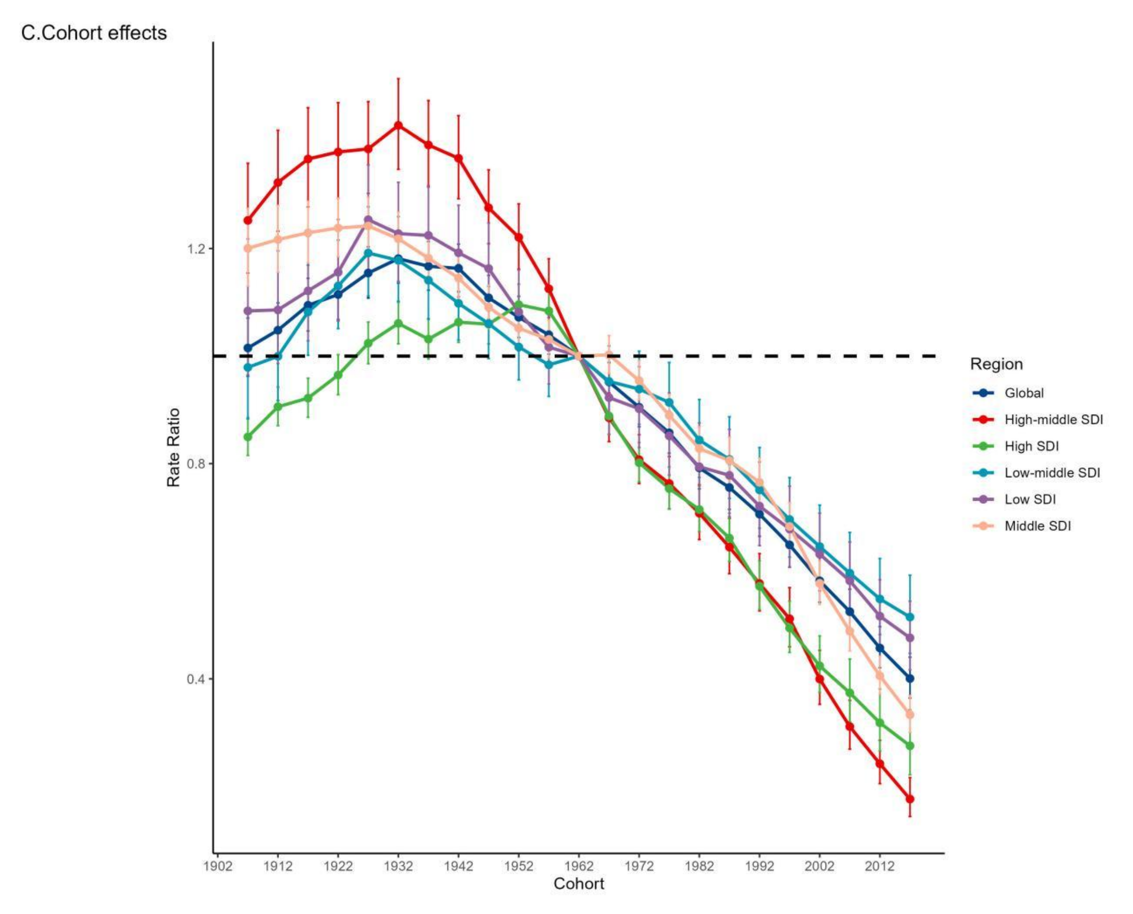
C.Cohort effects
Rate Ratio
1.2
0.8
0.4
1902
1912
1922
1932
1942
1952
1962
1972
1982
1992
2002
2012
Cohort
Region
Global
High-middle SDI
High SDI
Low-middle SDI
Low SDI
Middle SDI

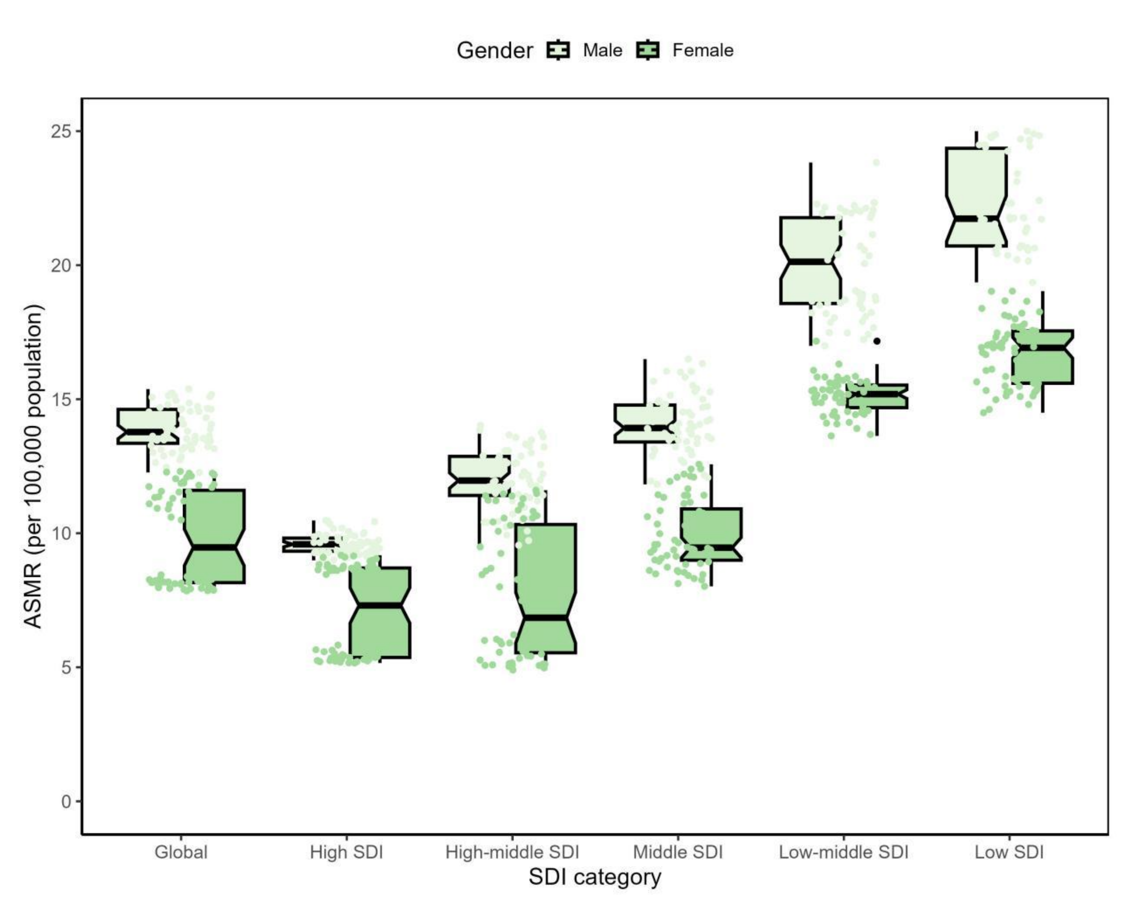
Gender
Male
Female
ASMR (per 100,000 population)
25
20
15
10
5
0
Global
High SDI
High-middle SDI
Middle SDI
Low-middle SDI
Low SDI
SDI category

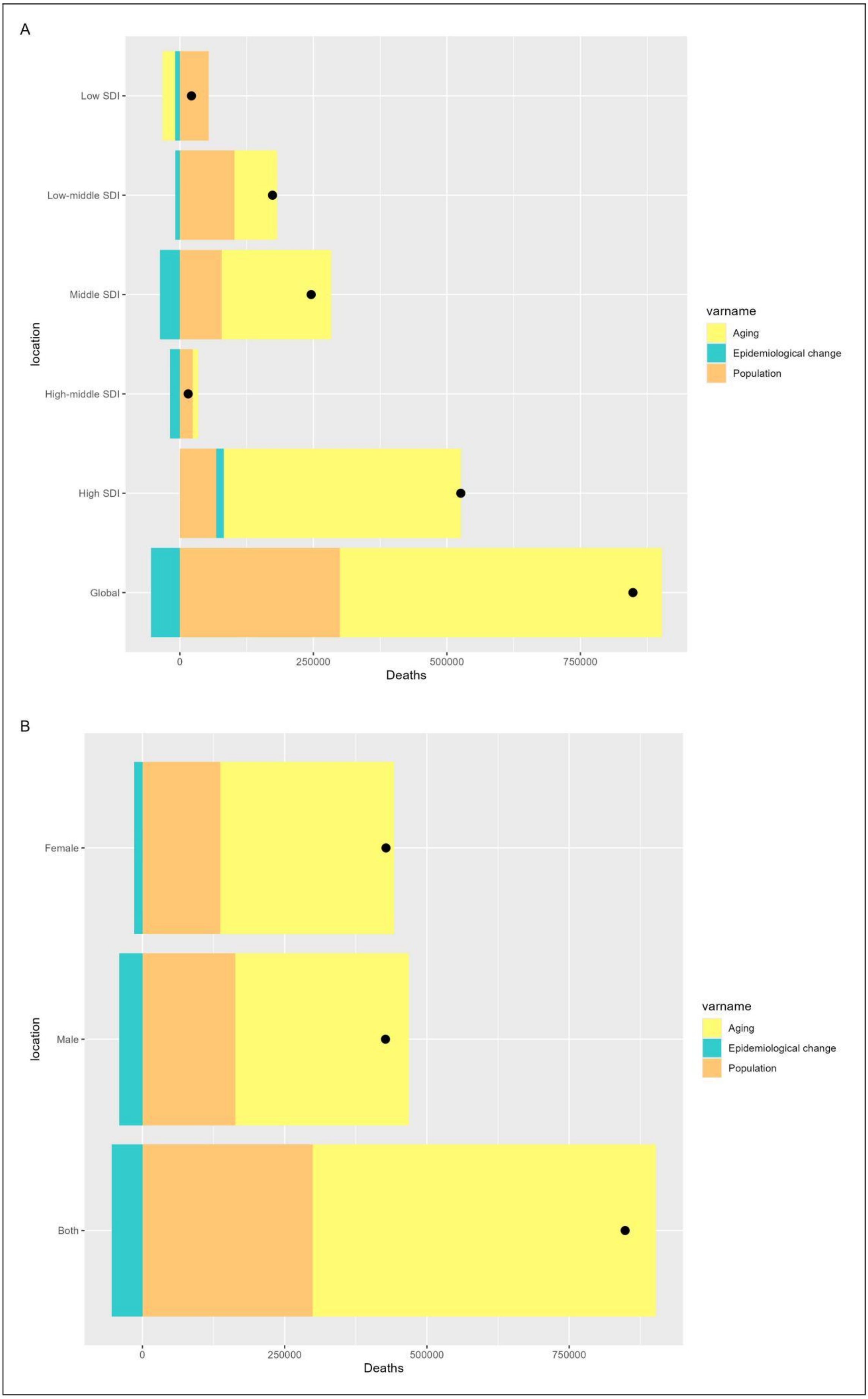
A
Low SDI
Low-middle SDI
Middle SDI
High-middle SDI
High SDI
Global
location
0
250000
500000
750000
Deaths
varname
Aging
Epidemiological change
Population
B
Female
Male
Both
location
0
250000
500000
750000
Deaths
varname
Aging
Epidemiological change
Population

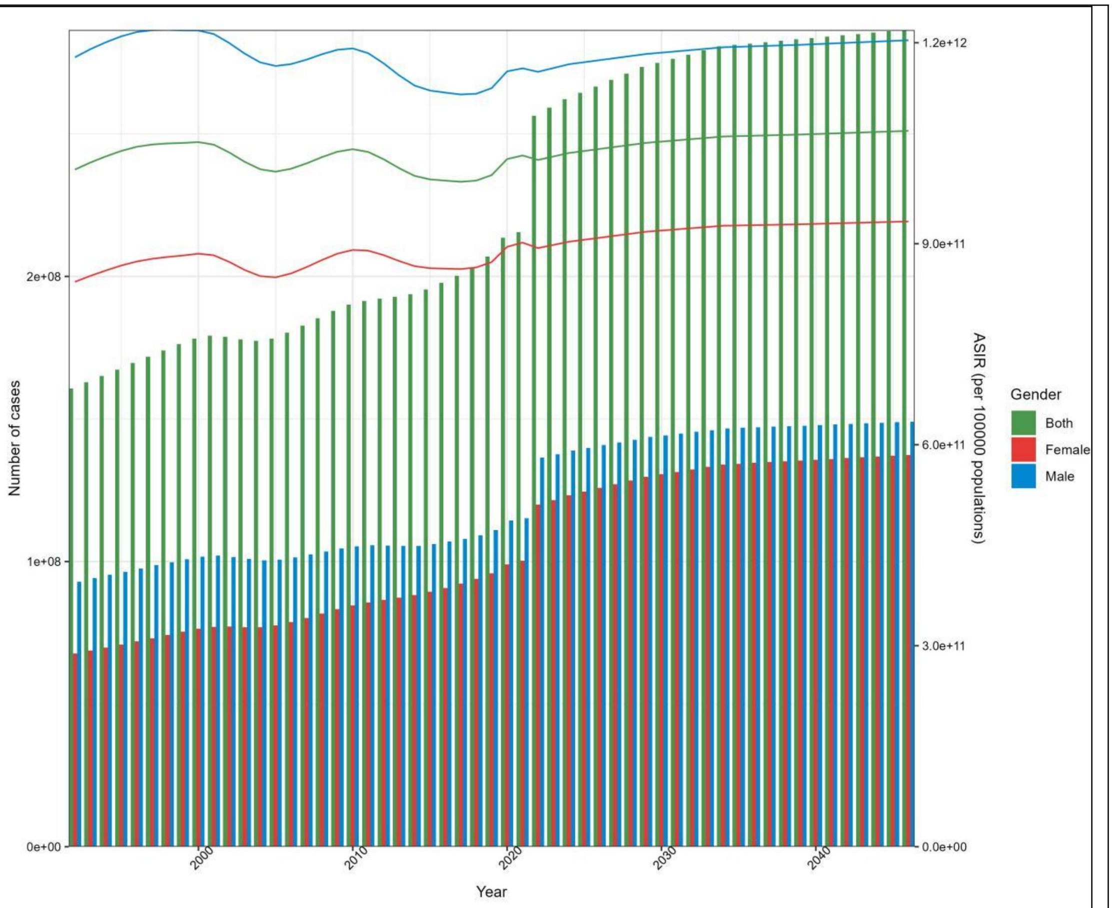
Number of cases
0e+00
1e+08
2e+08
2000
2010
2020
2030
2040
Year
ASIR (per 100000 populations)
0.0e+00
3.0e+11
6.0e+11
9.0e+11
1.2e+12
Gender
Both
Female
Male

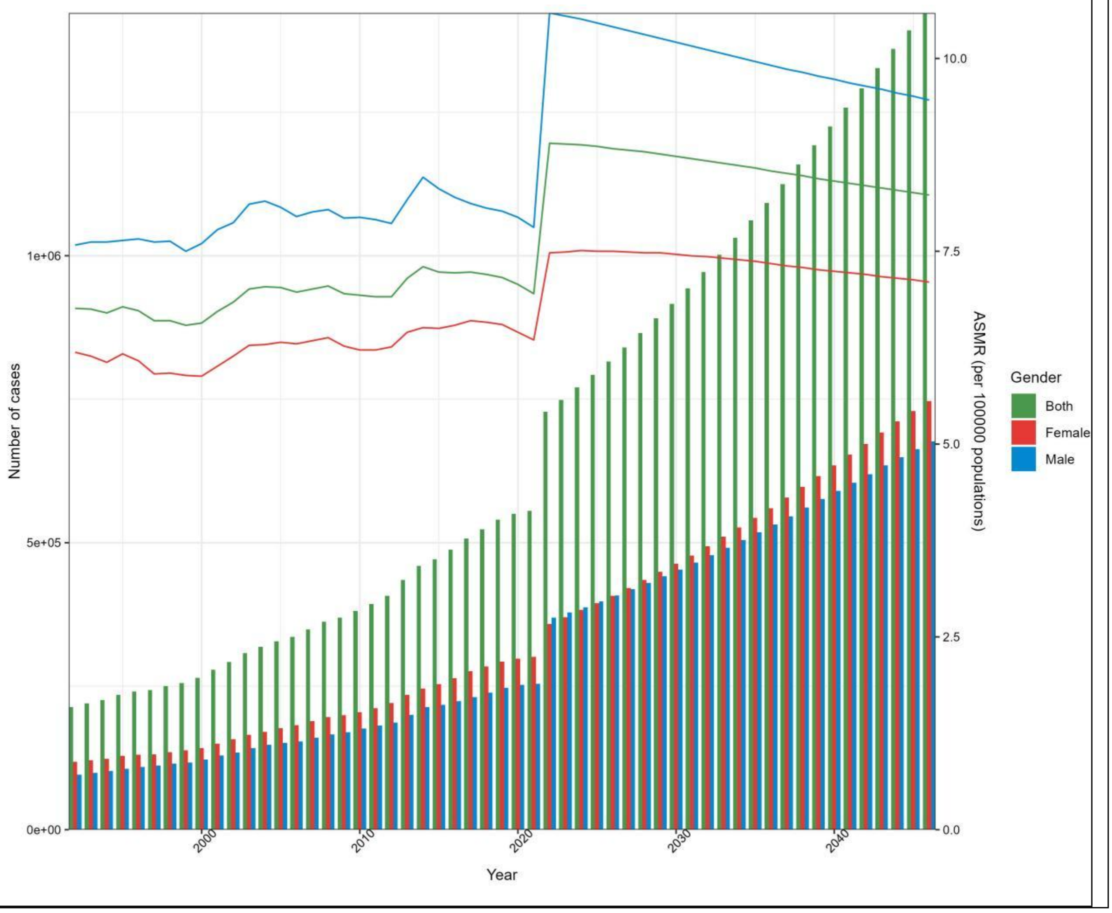
Number of cases
0e+00
5e+05
1e+06
2000
2010
2020
2030
2040
Year
ASMR (per 100000 populations)
0.0
2.5
5.0
7.5
10.0
Gender
Both
Female
Male

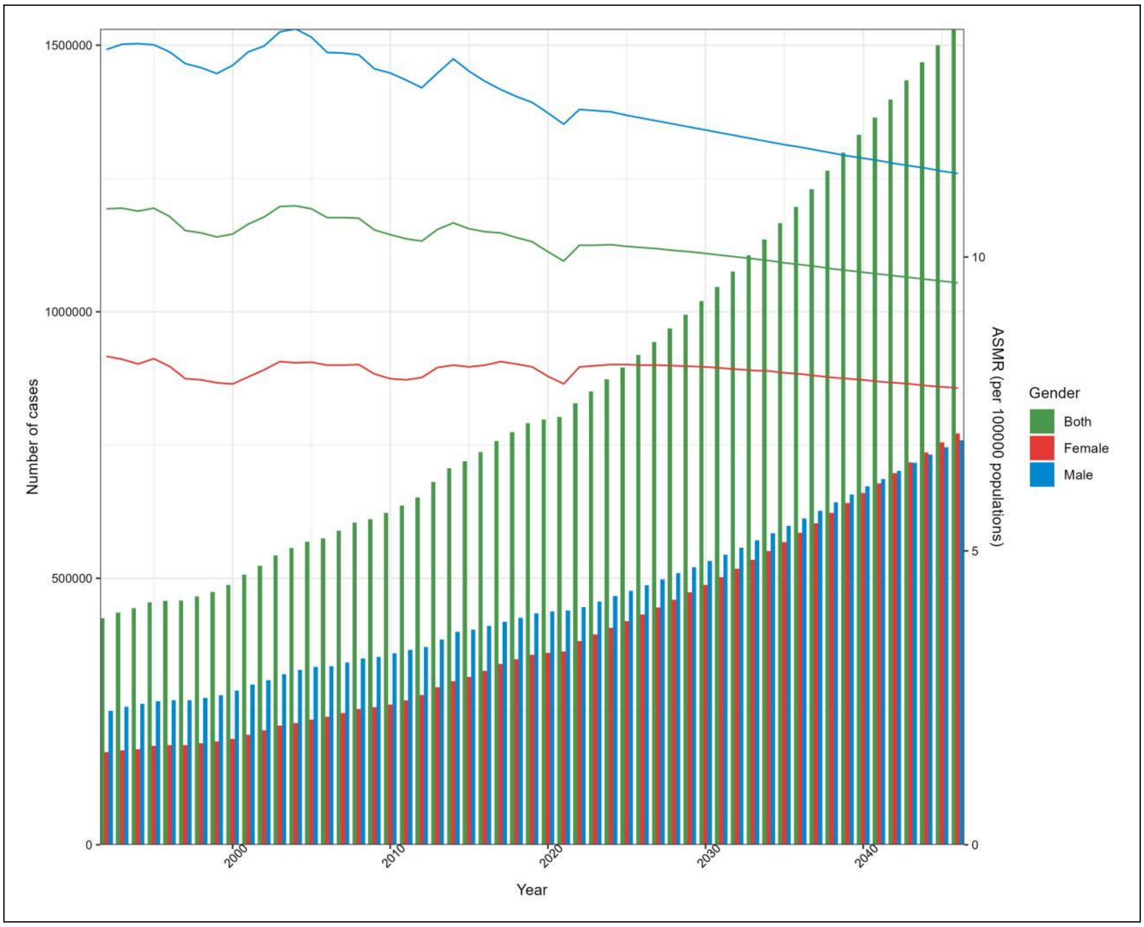

1500000
1000000
500000
0
Number of cases
2000
2010
2020
2030
2040
Year
10
5
0
ASMR (per 100000 populations)
Gender
Both
Female
Male
